# SensReg: A Regulon-Based Predictor of Neoadjuvant Chemotherapy Response in ER-Positive/HER2-Negative Breast Cancer

**DOI:** 10.21203/rs.3.rs-11001123/v1

**Published:** 2026-10-28

**Authors:** Bashir Lawal, Xiaosong Wang

**Affiliations:** University of Pittsburgh

**Keywords:** Neoadjuvant chemotherapy, SensReg, Transcription factor regulons, ER-positive/HER2-negative breast cancer, Pathological complete response, Precision oncology

## Abstract

Pretreatment predictors of neoadjuvant chemotherapy (NAC) response are lacking, especially in ER-positive/HER2-negative breast cancer, where cytotoxic benefit is most uncertain. From 1,158 transcription-factor regulons across five discovery cohorts, we derived SensReg, a locked 13-regulon logistic signature of chemotherapy sensitivity applied without refitting. SensReg was evaluated across 40 chemotherapy-only NAC datasets (n = 3,852, mean AUROC = 0.72), of which 39 (n = 3,840) were meta-analyzable (pooled AUROC 0.72, 95% CI 0.69–0.75), including 35 validation datasets independent of discovery (n = 3,092), of which 34 (n = 3,080, mean AUROC = 0.71) contributed to the pooled validation meta-analysis (pooled AUROC 0.70, 95% CI 0.67–0.73), and 20 HER2-antibody treatment arms from 17 series (pooled AUROC 0.66, 95% CI 0.60–0.72), outperformed 17 established signatures, and showed the strongest independent association with response after multivariable adjustment, the only signature significant at BH-FDR < 0.001 (adjusted OR 1.81, 95% CI 1.57–2.09; BH-FDR = 6.3×10^−15^), although this mutually adjusted comparison is exploratory because of limited events relative to the number of predictors. It provided positive estimated net benefit in ER-positive/HER2-negative disease (22 cohorts, n = 1,228) and retained discrimination within the recurrence-score intermediate stratum (AUROC 0.735). SensReg shows no detectable prognostic signal in a treatment-naive cohort but was associated with endocrine resistance and was carried mainly by malignant epithelium within immune-engaged microenvironments, associating with favorable chemotherapy-treated outcomes. SensReg is an interpretable, transferable predictor of treatment response warranting prospective evaluation.

## Introduction

Breast cancer is the most diagnosed malignancy and the leading cause of cancer death in women, with an estimated 2.3 million new cases worldwide in 2022 [1]. Neoadjuvant chemotherapy (NAC) is now a standard component of treatment for locally advanced and high-risk early breast cancer, and pathological complete response (pCR) after NAC is strongly associated with improved event-free and overall survival, most robustly in triple-negative (TNBC) and HER2-positive disease [2, 3]. This prognostic value has made pCR an accepted trial endpoint and a basis for post-neoadjuvant risk stratification: patients with pCR generally have favorable outcomes, whereas those with residual disease, quantified by the residual cancer burden index, carry higher long-term risk and may be candidates for treatment intensification [3, 4].

Despite this framework, response to NAC remains difficult to predict before treatment, and the clinical need for a pretreatment predictor is greatest precisely where response is least certain. In TNBC and HER2-positive disease, pCR rates are relatively high, although treatment paradigms differ by subtype. In early TNBC, adding pembrolizumab to neoadjuvant chemotherapy improved pCR and long-term outcomes in KEYNOTE-522 [5, 6], whereas HER2-positive disease is treated with HER2-directed therapy combined with chemotherapy. In ER-positive, HER2-negative breast cancer, by contrast, pCR rates are low and the benefit of cytotoxic chemotherapy is heterogeneous, so a pretreatment response predictor could help identify tumors with greater likelihood of chemosensitivity. Existing multigene assays only partially address this gap.

Prognostic signatures such as the 21-gene recurrence score (Oncotype DX) and the 70-gene signature (MammaPrint) were developed to estimate recurrence risk and adjuvant chemotherapy benefit rather than neoadjuvant response, and their ability to discriminate pCR varies by receptor subtype [7, 8]. Immune-based markers show the opposite subtype dependence: higher tumor-infiltrating lymphocyte (TIL) levels predict chemotherapy response and confer a survival benefit in TNBC and HER2-positive disease, but their prognostic direction differs in luminal breast cancer, indicating a distinct biology of the immune infiltrate across subtypes [9]. A predictor that captures a chemotherapy-sensitivity program with well-characterized behavior across receptor subtypes therefore remains an unmet need.

Transcription factor (TF) regulon activity offers a biologically anchored alternative to gene-level signatures. Rather than summing the expression of individual genes, regulon-based methods such as VIPER infer the activity of a regulatory program from the coordinated expression of a TF's direct target genes, using curated regulon resources such as DoRothEA, yielding estimates that are more stable across platforms and sample types [10, 11].

Cell-cycle TF programs are attractive candidates for chemotherapy-response prediction because cytotoxic agents preferentially affect proliferating cells; consistent with this, a target-gene signature of the cell-cycle repressor E2F4 predicts NAC response across breast cancer cohorts, although, like the genomic-prognostic assays, it performs better in ER-positive than ER-negative tumors [12]. Whether a multi-TF regulon model can improve on single-program signatures, remain independent of generic proliferation, and behave predictably across receptor subtypes and treatment contexts has not been established.

Here we derive and lock a 13-regulon logistic signature, SensReg, for neoadjuvant chemotherapy sensitivity, using five discovery cohorts and evaluating it without refitting across 40 chemotherapy cohorts (35 of them independent of discovery) and 20 HER2-antibody neoadjuvant treatment arms, 13 endocrine-therapy cohorts, single-cell and spatial transcriptomics, and institutional validation in the UPMC TNBC cohort. We show that SensReg predicts neoadjuvant response across chemotherapy and HER2-antibody settings, outperforms established prognostic and proliferation signatures, adds independent value in multivariable models, provides clinical net benefit in the ER-positive/HER2-negative setting of greatest decision uncertainty, and is carried mainly by the malignant compartment, which contributes 48% of SensReg-high cells, yet associates with an immune-engaged microenvironment and favorable outcome in TNBC.

## Results

### Derivation of a regulon signature of neoadjuvant chemotherapy sensitivity

To identify regulatory programs associated with chemotherapy response, we inferred 1,158 transcription-factor regulons across five NAC discovery cohorts (GSE194040, GSE23988, GSE25055, GSE25065 and GSE28844) and selected those with AUROC > 0.50 in every cohort, mean AUROC ≥ 0.70 and BH-FDR < 0.01. Thirteen regulons met these criteria (E2F1, E2F2, E2F3, E2F4, E2F8, TFDP1, MYCN, ZNF695, ZNF492, ARNTL2, CSRNP1, PIN1 and NKX3–1; Fig. 1a,b) and were combined in a multivariable logistic model whose fixed coefficients define the SensReg score (Fig. 1c). E2F3, CSRNP1 and ZNF492 carried the largest positive coefficients; E2F3 was the largest overall (+ 0.51), whereas E2F8 had the largest negative coefficient (− 0.47). In the discovery data, SensReg had a higher mean AUROC than any constituent regulon (0.81 versus 0.70–0.78; Fig. 1d), although this comparison is descriptive because the composite was fitted in the same cohorts. The regulons shared target genes and correlated activities (**Supplementary Fig. S1a,b**), and individual coefficient signs were therefore not interpreted mechanistically. Consistent with this redundancy, leave-one-regulon-out analysis changed mean discovery AUROC by < 0.006 for every regulon (**Supplementary Fig. S1c**).

To test specificity beyond discovery-based selection, we compared the mean validation AUROC of the 13 selected SensReg regulons with 10,000 random 13-regulon sets in the 35 held-out validation cohorts (Fig. 1e). The observed mean per-regulon AUROC was 0.659 and exceeded all 10,000 random 13-regulon sets drawn either from all 1,145 non-selected regulons (null mean 0.484, 95% range 0.452–0.519, maximum 0.557) or from 118 sensitivity-matched regulons defined by discovery mean AUROC 0.60–0.70 (null mean 0.574, 95% range 0.545–0.602, maximum 0.624). No random set reached the observed SensReg value in either comparison (empirical P < 0.0001; 0/10,000 exceedances). The SensReg score was subsequently evaluated without refitting across independent bulk, single-cell and spatial datasets (Fig. 1f).

### SensReg captures a distinct proliferation-linked regulatory program

Across normal GTEx tissues and single-cell lineages, SensReg activity was highest in proliferative and germline compartments (**Supplementary Fig. S2**), consistent with a physiological proliferation-linked program. As a positive control, the combined target set of the 13 regulons was most enriched in the E2F targets and G2M checkpoint Hallmark sets (Fig. 2a), an expected result rather than an independent finding, because six of the 13 regulons are E2F-family members or TFDP1, the E2F dimerisation partner, so their combined target set overlaps these Hallmark sets by construction; the test is one of gene-set membership and uses no tumor expression data. The targets resolve into an interconnected cell-cycle/E2F and DNA-repair network (**Supplementary Fig. S3**). To test whether these associations merely reflect bulk proliferation, we adjusted for two references of differing granularity: the single marker MKI67 and a multi-gene cell-cycle score. The multi-gene score, collinear with proliferation, drove the canonical cell-cycle associations negative (E2F targets, G2M checkpoint, MYC targets V1, mitotic spindle, DNA repair), the expected consequence of adjusting for a strongly collinear proliferation proxy, whereas MKI67 adjustment only attenuated them, leaving these pathways positively associated. Associations with non-proliferative hallmarks were robust to both: inflammatory response and hypoxia were retained, and the p53-pathway association was strengthened after adjustment (Fig. 2b). Consistently, in a correlation matrix against 17 reference variables, SensReg did not join the tight proliferation block. SensReg correlated only moderately with the proliferation signatures (Spearman ρ 0.53–0.67) and with MKI67 (ρ = 0.54), well below the correlations those signatures show with one another (ρ 0.91–0.99) or with MKI67 (ρ 0.85–0.90), and it was unrelated to nucleotide excision repair (ρ = 0.02, p = 0.51). It also diverged in direction: SensReg was positively correlated with EMT, stromal and myeloid programs (ρ 0.11–0.25) for which every proliferation signature, MKI67 included, was negatively correlated. In the variable PCA, SensReg carried the smallest loading on Dimension 1 of any proliferation-associated variable (Dimension 1, 55.2%; Dimension 2, 22.4%), and on Dimension 5 (4.1% of variance) it loaded + 0.69 against negative loadings for six of the eight comparators, including MKI67 (− 0.32) and Pan-F-TBRS (− 0.60) (Fig. 2c,d). SensReg is therefore proliferation-associated but remains distinct from the individual proliferation metrics evaluated here.

### SensReg predicts neoadjuvant treatment sensitivity across chemotherapy and HER2-antibody cohorts

We evaluated SensReg in 35 independent chemotherapy-only NAC validation datasets (n = 3,092) separate from the five discovery cohorts. Thirty-four of these datasets (n = 3,080) met the prespecified criteria for meta-analysis and yielded a random-effects pooled AUROC of 0.70 (95% CI 0.67–0.73; I^2^ = 43%); the mean per-dataset AUROC across the independent validation datasets was 0.71 (Fig. 3a). For descriptive analysis of the complete evidence set, inclusion of the five discovery cohorts yielded 40 datasets (n = 3,852) displayed in the forest plot. One dataset (GSE28796, n = 12) had a single responder and met the prespecified exclusion criterion of fewer than three samples in an outcome class; accordingly, the pooled AUROC of 0.72 (95% CI 0.69–0.75; I^2^ = 55%; permutation p < 5×10^−4^) was estimated from 39 meta-analyzable datasets (n = 3,840) (Fig. 3a,c). The signal extended to HER2-antibody-based neoadjuvant therapy across 20 treatment arms from 17 series (n = 734) (mean AUROC 0.67; pooled AUROC 0.66, 95% CI 0.60–0.72; I^2^ = 42%; permutation p < 5×10^−4^; Fig. 3b,c). Stratifying by receptor subtype revealed that predictive value was concentrated in ER-positive disease. In chemotherapy-only NAC cohorts, per-dataset mean AUROC was highest in ER+/HER2 − tumors (0.78) and lowest in ER−/HER2 + tumors (0.58), with intermediate performance in ER−/HER2− (0.64) and ER+/HER2+ (0.67) (Fig. 3d). The same ER dependence held in trastuzumab-based therapy: mean per-arm AUROC was 0.75 in ER+/HER2 + tumors versus 0.58 in ER−/HER2 + tumors (Fig. 3e; BH-FDR = 0.014). The subtype-resolved analysis was consistent: discrimination tracked ER-positive biology, with higher discrimination in ER+/HER2 + trastuzumab-treated disease (pooled AUROC 0.75) than in ER−/HER2 + disease (pooled 0.55, 95% CI 0.473–0.618, an interval that includes 0.5; the unweighted per-arm mean for this subgroup is 0.58), and intermediate discrimination in TNBC neoadjuvant chemotherapy (0.66, 95% CI 0.628–0.691). SensReg was therefore not distinguishable from chance in the ER−/HER2 + subgroup (**Supplementary Fig. S4**).

### SensReg outperforms established signatures and adds independent predictive value

Against 17 reference prognostic and proliferation signatures across the 34 evaluable validation datasets, excluding the five discovery cohorts in which SensReg was derived, SensReg had the highest median per-dataset AUROC (0.685). Signature orientation was fixed once from the discovery cohorts rather than re-set in each dataset, so a signature that reverses direction between cohorts is shown doing so; on that basis SensReg was also the most directionally stable signature, exceeding 0.50 in all 34 evaluable validation datasets, whereas every comparator fell below 0.50 in at least one (median 8 of 34), and every pairwise comparison favored SensReg (two-sided paired Wilcoxon signed-rank test on the per-dataset AUROCs, all BH-FDR < 0.01; Fig. 4a). The composite also outperformed all 13 of its constituent regulons across the validation cohorts, with the highest mean per-dataset AUROC (0.706 versus 0.614–0.689 for the individual regulons; all 13 comparisons BH-FDR < 0.05, 11 of 13 at < 0.01, the exceptions being NKX3–1 (BH-FDR 0.029) and E2F3 (0.047); Supplementary Fig. S5). Adding SensReg to each comparator improved discrimination in every case (ΔAUROC 0.058–0.170; all 17 comparisons BH-FDR < 0.001; Fig. 4b). Within each cohort both scores were standardized to unit SD and summed with equal weight, so the combination uses no outcome information and no weights are fitted; the resulting increments are nonetheless apparent rather than cross-validated. Their ranking is largely a function of how weak each comparator is: the increment correlates with the comparator's own AUROC at r = − 0.98, and is predicted almost exactly (r = 0.99) by assuming that adding SensReg simply recovers SensReg's own performance. Consistent with that, no combination outperformed SensReg used alone, so the gain reflects replacing a weaker score rather than genuine complementarity. A cross-validated increment over a different reference, specifically an age-and-grade clinical model rather than a comparator signature, is reported separately as ΔAUROC 0.16 (Fig. 5a) and is not directly comparable with these in-sample values. Most importantly, in a random-effects meta-analysis of dataset-specific, fully adjusted multivariable logistic models, SensReg showed the strongest independent association with pCR after mutual adjustment (pooled adjusted OR 1.81, 95% CI = 1.57–2.09; BH-FDR = 6.3 × 10^−15^), the only signature significant at BH-FDR < 0.001 and by more than twelve orders of magnitude; three comparators also remained significant after BH correction at FDR < 0.05 (MDSC consensus signature, BH-FDR 0.020; E2F targets, 0.023; MKI67, 0.045), so SensReg is the dominant but not the sole independent predictor in this model (Fig. 4c).

### SensReg provides positive estimated clinical net benefit in ER-positive/HER2-negative disease

ER+/HER2 − breast cancer is the setting of greatest clinical uncertainty regarding neoadjuvant chemotherapy, given its low pCR rate. Adding SensReg to an age-and-grade model improved pCR discrimination across the five ER+/HER2 − cohorts with complete age, grade, SensReg score and pCR for every patient (GSE23988, GSE25055, GSE63471, GSE140494 and GSE163882). These were the only five cohorts meeting that requirement and all five were used; two of them, GSE23988 and GSE25055, are discovery cohorts, so this panel is not independent of derivation. The pooled estimate was a positive ΔAUROC 0.162 (95% CI 0.002–0.323; P = 0.047; I^2^=75.7%, Fig. 5a), and SensReg remained independently associated with pCR after adjustment for age and grade (all per-cohort odds ratios > 1; pooled estimate excluding 1; **Supplementary Fig. S6**). Given this comparatively strong performance in ER+/HER2 − disease, we evaluated this subgroup across 22 cohorts comprising 1,228 patients: the pooled AUROC was 0.77 (95% CI 0.719–0.819; I^2^ = 28%), with a mean dataset-specific AUROC of 0.78 and an overall pCR rate of 11.6% (Fig. 5b). Four of these 22 cohorts (410 of 1,228 patients) are discovery cohorts; restricting to the 18 validation cohorts (n = 818, 92 pCR, prevalence 11.2%) gives a pooled AUROC of 0.77 (95% CI 0.70–0.82; I^2^ = 23%) and a mean of 0.78, so removing the derivation cohorts does not change the estimate. Decision-curve analysis showed positive net benefit for SensReg-guided prediction across the clinically relevant threshold range, where neither the treat-all nor treat-none default strategy is informative. In this low-prevalence subgroup, the treat-all strategy became net-harmful once the decision threshold exceeded the pCR prevalence (11.6%), an arithmetic property of any low-prevalence setting rather than a property of SensReg, whereas SensReg-guided prediction maintained positive net benefit: 0.074 at a 5% threshold, 0.055 at 10%, 0.037 at 15% and 0.023 at 20%, against 0.070, 0.018, − 0.040 and − 0.104 for treat-all at the same thresholds. A net benefit of 0.055 at a 10% threshold corresponds to 5.5 net true-positive equivalents per 100 patients relative to treat-none, after accounting for the weighted harm of false-positive treatment. SensReg exceeded both default strategies from roughly 4% to approximately 33% and was indistinguishable from treat-none above that (Fig. 5c). These findings support potential incremental clinical value but require prospective evaluation before clinical implementation. Notably, SensReg showed no significant prognostic association in treatment-naive disease: in an untreated TCGA cohort (n = 292), it did not discriminate progression-free interval (AUROC 0.580; HR 1.22, 95% CI 0.72–2.07; p = 0.450) or overall survival (AUROC 0.515; HR 1.06, 95% CI 0.70–1.60; p = 0.789; Supplementary Fig. S7), supporting a treatment-response rather than intrinsic-prognostic signal.

### SensReg retains predictive value within the research-defined recurrence-score intermediate-risk gray zone in ER+/HER2 − breast cancer

We next asked whether SensReg provides predictive information beyond a research-based approximation of the 21-gene recurrence score (RS; Methods). Across 19 ER+/HER2 − cohorts with computable RS (n = 910; 118 pCR), SensReg and RS were each strongly associated with pCR in univariable models (SensReg OR 2.76 per 1-SD, 95% CI 2.20–3.45, p = 8.6×10^−19^; RS OR 2.72, 95% CI 2.15–3.43, p = 6.1×10^−17^). In a joint model, SensReg remained independently associated with pCR after adjustment for RS (OR 1.96 per 1-SD, 95% CI 1.49–2.58, p = 1.3×10^−6^), while RS also retained independent association after adjustment for SensReg (OR 1.78, 95% CI 1.35–2.35, p = 5.4×10^−5^), indicating complementary rather than redundant information (Fig. 5d). Adding SensReg to RS improved pooled discrimination, estimated on the full data rather than out of fold, from AUROC 0.746 to 0.781 (+ 0.035), while adding RS to SensReg raised it from 0.767 to 0.781 (+ 0.014) (likelihood-ratio test p = 8.9×10^−7^; **Supplementary Fig. S8**).

We then evaluated SensReg within research-defined RS-intermediate groups, where the escalation decision is least certain [13, 14]. In this intermediate stratum (cohort median ± MAD; patient-level pool of 678 intermediate-stratum patients, 62 pCR, across 19 ER+/HER2 − cohorts, with an estimable per-cohort AUROC in 12 of these cohorts), SensReg separated responders from non-responders with a pooled AUROC of 0.735 (95% CI 0.659–0.811, Fig. 5e) and an OR of 2.67 per 1-SD (1.93–3.70, p = 3.73×10^−9^; Supplementary Table S1), and the result was consistent across the quartile (0.722) and research-calibrated 11–25 (0.700) definitions. This intermediate band was itself an ER+/HER2 − phenomenon: on the calibrated scale ER+/HER2 − tumors distributed approximately 25% low, 50% intermediate and 25% high, whereas intermediate-range tumors were less common in HER2-positive and triple-negative disease, which were predominantly high-risk (ER+/HER2 + 57% and triple-negative 71%; the ER−/HER2 + group, shown separately in the panel, is higher still) (Fig. 5f), supporting restriction of the recurrence-score comparison to ER+/HER2−.

### SensReg marks endocrine resistance across treatment settings

We applied the SensReg score, without refitting, to endocrine-treated breast cancer cohorts. Across nine adjuvant cohorts with survival data (n = 2,351), higher SensReg activity (above the within-cohort mean locked score) was associated with worse outcome in every cohort by point estimate (per-cohort HRs 1.11–4.26), though most individual confidence intervals were non-significant. By random-effects meta-analysis, the association held for overall survival (5 cohorts; HR 1.35, 95% CI 1.08–1.70; p = 0.009; I^2^ = 0%; **Supplementary Fig. S9a**) and for disease-related events (recurrence- and metastasis-free survival; 4 cohorts; HR 1.86, 95% CI 1.01–3.41; p = 0.047; I^2^ = 47%; **Supplementary Fig. S9b**). The two endpoint families are reported separately and are not pooled, because overall survival and recurrence- or metastasis-free survival are not exchangeable endpoints. In four additional cohorts with binary endocrine outcome or response annotations spanning the adjuvant (GSE16391; GSE82172) and neoadjuvant (GSE5462, GSE145325) settings, SensReg separated endocrine-resistant from endocrine-sensitive tumors in the same direction in all four (AUROC 0.62–0.70; pooled 0.66, 95% CI 0.56–0.75, p = 0.002, I^2^ = 0%), reaching nominal significance in one (GSE145325, p = 0.016) and BH-FDR significance in none; three of the four confidence intervals include 0.5 (**Supplementary Fig. S9c**), with directionally consistent estimates across the adjuvant and neoadjuvant settings and across letrozole and tamoxifen; the number of cohorts is too small for a formal test of interaction, so consistency across drug and setting is suggested rather than established.

### The malignant compartment contributes most SensReg-high cells, in T-cell- and macrophage-engaged microenvironments

We next examined the cellular distribution of SensReg using breast cancer single-cell RNA-sequencing data from 13 patients. The malignant compartment was the largest single source of SensReg-high cells, contributing a higher fraction of each tumor's cells as SensReg-high than any other compartment (paired Wilcoxon signed-rank versus malignant, BH-corrected across the seven compartments plotted; significant for dendritic, endothelial, fibroblast, macrophage and B-cell compartments at FDR < 0.05, and in the same direction but non-significant for T-cell and NK-cell compartments), with detectable activity also in T-cell and macrophage compartments (Fig. 6a–c). Patient-level analysis showed interpatient variation in the relative contribution of these compartments to the SensReg-high population (Fig. 6a). Compartment-resolved marker analyses further associated SensReg-high populations with macrophage inflammatory programs and antigen-presentation machinery: in SensReg-high macrophages 8 of the 12 M1 pro-inflammatory markers were higher (IL1B, TNF, IL6, CXCL9, CXCL10, CXCL11, CD80 and STAT1) while the M2 markers CD163 and CHI3L1 and the pan-macrophage identity markers CD14 and CD68 were lower, and in malignant cells all seven antigen-presentation genes were higher, and with a T-cell phenotype that combines effector features with uniformly higher checkpoint expression. Within the T-cell compartment (2,898 cells, 705 SensReg-high), all seven checkpoint/exhaustion markers were higher in SensReg-high cells (PDCD1, CTLA4, LAG3, HAVCR2, TIGIT, TOX and ENTPD1; all BH-FDR < 1 × 10^−7^) while all five naive/memory markers were lower (CCR7, IL7R, SELL, TCF7 and LEF1), alongside higher GZMB, PRF1 and IFNG (Supplementary Fig. S10). These are cell-level comparisons that treat every cell as an independent observation, and we therefore repeated them at patient level, each patient contributing one paired difference between the mean of its SensReg-high and SensReg-low cells. On that basis the macrophage findings are partly supported: IL1B and CXCL10 remain higher and CD163, CD14 and CD68 lower in SensReg-high macrophages (11 patients; BH-FDR 0.022 each), while the remaining markers do not reach significance. The T-cell panel becomes uninformative because only 6 of the 13 patients contribute enough T cells in both groups, although the direction is consistent across those patients (IFNG higher in 6 of 6; CCR7, IL7R and TCF7 lower in 6 of 6). The malignant antigen-presentation result does not survive: with all 13 patients contributing, six of the seven genes reverse direction within patients, and the pooled association reflects between-patient composition rather than a within-tumor contrast, because patients with a larger SensReg-high fraction also have higher antigen-presentation expression overall (for HLA-A, ρ = 0.51 across patients; only 5 of 13 patients show a positive within-patient difference). We therefore report the compartment-resolved marker phenotypes as descriptive and do not claim a within-tumor antigen-presentation effect. This is a differentiated, checkpoint-high rather than a simply activated T-cell phenotype, and its functional significance is untested here. These analyses nonetheless indicate that SensReg activity is not restricted to malignant-cell proliferation. The compartment ratio plotted in Fig. 6a is expressed per sample as a fraction of all cells in that sample, so it reflects both the per-cell frequency of SensReg-high cells and the abundance of the compartment. Malignant cells accounted for 48.4% of SensReg-high cells while making up 50.7% of all cells profiled, so they were the dominant source of SensReg-high cells without being enriched for them (enrichment 0.95); on a per-cell basis the SensReg-high frequency was 18.7% in malignant cells, 36.8% in macrophages and 3.8% in fibroblasts (enrichment 1.87 and 0.19 respectively). SensReg activity is therefore carried predominantly, but not selectively, by the malignant compartment.

Across intrinsic molecular subtypes, SensReg-positive tumors were most frequent in Basal and HER2-enriched disease (Fig. 7a). Consistent with the single-cell findings, SensReg was weakly but consistently associated with tumor-infiltrating lymphocyte fraction in TCGA-BRCA, accounting for about 6% of the variance in TIL fraction (ρ = 0.24, p = 3.12×10^−14^; n = 949) and inversely associated with estimated tumor purity (ρ = −0.25, p = 4.64×10^−17^; n = 1,065) (Fig. 7b,c). By CIBERSORT deconvolution (n = 1,104), SensReg correlated positively with undifferentiated (M0) and M1 macrophages (ρ = 0.33 and 0.32), activated CD4 + memory (ρ = 0.33), follicular-helper (ρ = 0.29) and CD8 + T cells (ρ = 0.25), and negatively with monocytes, activated mast cells and eosinophils (ρ = −0.12 to − 0.07). Six further positive associations were also significant and are reported here for completeness: naive B cells (ρ = 0.19), plasma cells (ρ = 0.18), resting CD4 + memory T cells (ρ = 0.17), regulatory T cells (ρ = 0.15), activated myeloid dendritic cells (ρ = 0.13) and M2 macrophages (ρ = 0.10). Because SensReg is itself inversely associated with tumor purity, these correlations were repeated as partial Spearman correlations adjusting for purity (n = 1,065). The five leading associations persisted with modest attenuation (M0 macrophages ρ 0.32 → 0.26, activated CD4 + memory 0.33 → 0.24, M1 macrophages 0.30 → 0.19, follicular-helper 0.28 → 0.22 and CD8 + T cells 0.24 → 0.13; all BH-FDR < 1 × 10^−4^), as did the TIL association (0.24 → 0.19), and 12 of 22 associations remained significant. The regulatory-T-cell, resting CD4 + memory and M2-macrophage associations did not survive purity adjustment, so we do not interpret them as evidence about the character of the accompanying infiltrate. In total 14 of 22 associations were significant at BH-FDR < 0.05, though effect sizes were modest (all |ρ| < 0.34), and the strongest single association is with M0 macrophages, an undifferentiated and proliferation-correlated CIBERSORT compartment whose deconvolution estimates are the least well identified of the 22 (Fig. 7d; **Supplementary Table S2**). Representative histology showed a cellular, TIL-rich SensReg-high tumor versus a fibrotic, immune-poor SensReg-low tumor (Fig. 7e).

### SensReg exhibits distinct spatial organization across tumor and microenvironmental states

Spatial transcriptomic analysis of the BCTL TNBC cohort (n = 94) [15] showed non-random localization of SensReg-high regions across histopathological compartments. SensReg-high spots were most strongly enriched within tumor and in situ compartments (Fig. 8a). Spatial megacluster analysis further showed enrichment in five of the 14 megaclusters (MC1, MC2, MC3, MC5 and MC6), with MC1 far the strongest (patient-pooled odds ratio 4.07, 95% CI 2.63–6.28), followed by MC3 (2.91), MC5 (2.82), MC6 (2.31) and MC2 (2.02). MC1 is present in only five patients, so that leading estimate is fragile (Fig. 8b). Read against the megacluster annotations, all five enriched megaclusters carry a high-proliferation label, but their immune labels differ: MC1, MC2 and MC3 are annotated immune-depleted, MC5 immune-activated, and MC6 carries no immune term. Conversely three of the five immune-activated megaclusters (MC9, MC10 and MC14) were depleted of SensReg-high spots. Across the fourteen annotated megaclusters this association between immune annotation and enrichment is not statistically significant (Fisher exact p = 0.5804) and is reported as descriptive, but it indicates that the immune-rich association reflects a subset of niches rather than the dominant one. Consistent with this heterogeneity, spatial SensReg score was lowest in immune-deserted tumors by tumor-immune microenvironment classification (**Supplementary Fig. S11a**) and was depleted in the luminal androgen receptor (LAR) and mesenchymal stem-like (MSL) subtypes of the Bareche triple-negative classification, which are inherited from the Lehmann subtyping scheme [16], across the global and stroma pseudo-bulk (**Supplementary Fig. S11b**), consistent with its endocrine-resistance direction. Because the 199,634 spots are nested within 94 patients and 278 slides, histological enrichment was additionally estimated per patient and pooled by random effects rather than treating spots as independent. On that basis SensReg-high spots remained enriched in tumor regions (odds ratio 1.97, 95% CI 1.74–2.24; higher in 61 of 69 patients), whereas none of the immune compartments reached significance: lymphocyte 0.92 (0.75–1.15), high-TIL stroma 0.98 (0.59–1.62) and lymphoid nodule 0.51 (0.17–1.54), and between-patient heterogeneity was high throughout (I^2^ 60–95%). The spatial immune associations should therefore be read as descriptive. Spatial analysis relative to necrotic regions showed that SensReg activity peaked in a perinecrotic band and declined with distance from necrosis (**Supplementary Fig. S12**), although this gradient is also a pooled-spot result: across the 49 patients with evaluable necrotic regions the per-patient Spearman correlation was negative in 26 and positive in 23, with a median of only − 0.01 and no overall departure from zero (one-sample Wilcoxon p = 0.36); of the 32 patients reaching BH-adjusted significance, 18 were negative and 14 positive. The gradient is therefore a pooled-spot phenomenon and not a consistent within-tumor feature. Distance is expressed in spot pitches, one pitch corresponding to 19.87 image pixels; that spacing is constant across slides (coefficient of variation 0.29%), so distances are comparable between tumors even though the absolute micrometre scale depends on the array pitch of the source platform. Subject to these caveats the pooled pattern (Spearman ρ = −0.11 across 39,521 spots) is consistent with a proliferative, metabolically stressed niche. Together, these analyses indicate that SensReg is spatially associated with proliferative tumor regions while occurring across heterogeneous molecular, immune and histopathological microenvironments.

We next examined clinical outcomes within the chemotherapy-treated subset (n = 83; n = 81 with available outcome data). SensReg-high tumors were associated with longer overall survival (HR 0.31, profile-likelihood 95% CI 0.10 to 0.89; Cox likelihood-ratio p = 0.030) and distant recurrence-free survival (HR = 0.29, profile-likelihood 95% CI 0.09 to 0.79; Cox likelihood-ratio p = 0.015) than SensReg-low tumors (Fig. 8c). Representative spatial maps illustrated dense, lymphocyte-rich SensReg-high tumors in long-term survivors versus stroma-dominant SensReg-low tumors in early deaths (Fig. 8d). Given the retrospective design and limited number of outcome events, these survival associations require independent validation.

### Institutional validation in a previously published UPMC TNBC cohort, independent of the discovery data

To validate SensReg in an independent institutional cohort, we applied the SensReg model to 48 chemotherapy-treated UPMC TNBC patients (of 67 patients with a SensReg score; GSE312235), of whom 44 had evaluable overall-survival follow-up and 45 had evaluable recurrence-free-survival follow-up. SensReg scores were lower in tumors that recurred during available follow-up than in those that did not (one-tailed Mann–Whitney p = 0.03; Fig. 9a), and observed recurrence was less frequent in SensReg-high than SensReg-low tumors (2/21, 9.5% versus 9/27, 33%; Fisher exact one-sided p = 0.052, consistent with the pre-specified direction that higher SensReg marks chemosensitivity; Fig. 9a). Follow-up is right-censored, with a median of 65 months (5.4 years) for recurrence-free survival, and only a minority of patients were observed to 10 years (44, 27 and 11 patients remained evaluable at 2, 5 and 10 years), so both panel-a comparisons are descriptive summaries of observed recurrence. However, censoring-aware comparison revealed that SensReg-high tumors had longer recurrence-free survival (HR = 0.25, 95% CI 0.04–0.99; Cox likelihood-ratio p = 0.047; Fig. 9b). In addition, the SensReg score discriminated survival with time-dependent AUCs of 0.77 (95% CI 0.55–0.95) at 2 years, 0.80 (95% CI 0.56–0.98) at 5 years and 0.82 (95% CI 0.55–1.00) at 10 years (Fig. 9c); the intervals are wide and the 10-year estimate rests on only 11 patients (7 events), so these values are hypothesis-generating. Given the retrospective design and small number of events, these associations are exploratory and hypothesis-generating and require prospective validation.

## Discussion

In this study we derived and locked SensReg in five discovery cohorts and subsequently evaluated it without refitting in 35 independent neoadjuvant chemotherapy validation cohorts, alongside HER2-antibody, endocrine-therapy, single-cell, spatial, and institutional analyses. SensReg predicted neoadjuvant response with a pooled AUROC of 0.72 in the chemotherapy setting and 0.66 in the HER2-antibody setting, outperformed 17 established prognostic and proliferation signatures, and showed the strongest independent association with pCR after mutual multivariable adjustment, although that comparison is exploratory, resting on a median of only 1.2 pCR events per predictor (pooled adjusted odds ratio 1.81, 95% CI 1.57–2.09). Its predictive value was concentrated in estrogen-receptor (ER)-positive disease, where it provided positive decision-curve net benefit. SensReg activity resolved into an interconnected cell-cycle, E2F and DNA-repair network and was highest in proliferative normal and germline compartments, consistent with a proliferation-linked program. Cytotoxic chemotherapy preferentially affects cycling cells, so a coordinated proliferation and replication-stress program is a mechanistically plausible determinant of chemosensitivity, and this rationale also underlies the earlier single-program E2F4 target signature that predicts pCR [12]. Importantly, SensReg was strongly proliferation-linked but was not identical to any single proliferation metric. Adjustment for a multigene cell-cycle score reversed the canonical cell-cycle pathway associations, consistent with strong collinearity and possible over-adjustment, whereas adjustment for MKI67 produced attenuation without the same reversal. We therefore do not interpret the cell-cycle-adjusted residual as proof of proliferation independence. Instead, the orthogonality analyses indicate that SensReg captures a correlated regulatory program distinct from MKI67 and from the proliferation modules of Oncotype DX and MammaPrint. Consistent with this distinction, SensReg retained an independent association with treatment response in multivariable models.

Inferring regulon activity with VIPER on curated DoRothEA regulons [10, 11], rather than summing raw gene expression, provides a platform-stable functional estimate that plausibly contributes to the transferability we observed across microarray and RNA-sequencing cohorts and across bulk, single-cell and spatial data. The receptor-subtype dependence of SensReg mirrors, but does not merely reproduce, that of existing assays. Oncotype DX and MammaPrint were developed primarily for recurrence-risk assessment, whereas the E2F4 signature has been evaluated for neoadjuvant pathological response[7, 8, 12]. SensReg shares the stronger ER-positive orientation reported for these signatures but improves on the reference panel in our head-to-head analysis across the 34 evaluable validation cohorts and retains independent association after multivariable adjustment. The immune infiltrate shows the opposite subtype behavior, with tumor-infiltrating lymphocytes (TILs) predicting chemotherapy response most strongly in triple-negative and HER2-positive disease [9]. SensReg and TIL-based markers show opposite subtype orientations and may capture different axes of chemosensitivity, though we did not fit a joint SensReg + TIL model and SensReg correlated positively with TILs (ρ = 0.24). The greatest translational value of SensReg lies precisely where clinical decisions are hardest. In ER-positive, HER2-negative disease the pCR rate is low (11.6% in our pooled ER-positive, HER2-negative analysis), the absolute benefit of cytotoxic chemotherapy is heterogeneous, and existing genomic assays were built to estimate adjuvant recurrence risk rather than neoadjuvant response. Landmark adjuvant trials established that many patients in this group can safely forgo chemotherapy [13], yet those trials do not directly inform who will respond if chemotherapy is given in the neoadjuvant setting.

Because pCR is associated with long-term outcome [2, 3] and residual disease burden stratifies post-neoadjuvant risk [4], a pretreatment predictor of response could inform prospective treatment-stratification strategies, including trials of alternative or de-escalated approaches for tumors predicted to have low chemosensitivity. However, prediction of pCR alone cannot establish absence of chemotherapy benefit. Decision-curve analysis is informative here: in a low-prevalence setting the default treat-all strategy becomes net-harmful once the decision threshold exceeds prevalence, whereas SensReg-guided prediction maintained positive net benefit across the clinically relevant threshold range (0.055 at a 10% threshold, equivalent to 5.5 net true-positive equivalents per 100 patients after accounting for the threshold-weighted harm of false-positive treatment; this estimate rests on cross-validated calibration of the locked score rather than on the locked score applied unchanged), indicating potential clinical utility beyond discrimination statistics alone [17]. This is also the subgroup least addressed by the immunotherapy era, since checkpoint blockade added to chemotherapy has transformed early triple-negative disease but has a more limited established role in luminal breast cancer [5, 6]. The recurrence-score analysis further suggests that SensReg captures information not contained within an established genomic-risk framework. In 19 ER+/HER2 − cohorts, SensReg and the research-based RS remained independently associated with pCR when modeled together, and adding SensReg to RS improved discrimination. Importantly, SensReg also retained predictive value within a cohort-defined intermediate RS range, where risk stratification by the recurrence score alone is less informative. Because the RS used here is a research approximation rather than the commercial Oncotype DX assay, these findings should not be interpreted as clinical validation in patients with measured intermediate Oncotype scores. Instead, they provide a rationale for prospective evaluation of SensReg in patients with clinically determined intermediate recurrence scores.

A notable and biologically coherent finding is the divergent behavior of SensReg across treatment modalities. The SensReg score marked chemotherapy sensitivity and was associated with endocrine resistance, being associated with worse survival across nine endocrine-treated cohorts and discriminating endocrine-resistant tumors across the adjuvant and neoadjuvant settings, while no prognostic signal was detected in untreated tumors, a comparison that is roughly eight-fold smaller and whose confidence interval includes the endocrine estimate. This pattern is consistent with the biology of proliferative luminal tumors, which tend to be more responsive to cytotoxic therapy yet less dependent on, and less durably controlled by, ER signaling. Clinically, this dual behavior motivates prospective testing of whether SensReg can identify ER-positive tumors with differential benefit from endocrine-based versus chemotherapy-containing strategies. The absence of a detectable prognostic signal in untreated TCGA-BRCA supports a treatment-associated rather than purely prognostic interpretation.

Single-cell and spatial analyses refined the interpretation of the score. SensReg activity was carried mainly by the malignant compartment, which supplied 48% of SensReg-high cells in proportion to its abundance rather than through enrichment, yet was found in immune-engaged microenvironments, correlating positively with TILs and inversely with tumor purity, and associating with M1 macrophage and CD8 and follicular-helper T-cell programs. The accompanying T cells were, however, checkpoint-high and naive/memory-low, and the deconvolution infiltrate included regulatory T cells and M2 macrophages, so the microenvironment is immune-engaged but not uniformly effector. Spatially, SensReg peaked in a perinecrotic, metabolically stressed niche when spots were pooled, though this gradient was not consistent across individual tumors, and was depleted in immune-deserted tumors and in the LAR and MSL subtypes, consistent with its endocrine-resistance direction. In the chemotherapy-treated triple-negative setting, higher SensReg marked tumors with favorable overall and distant recurrence-free survival in the spatial cohort, and with longer recurrence-free survival in the independent institutional series.

Together, these observations show that malignant-cell SensReg activity co-occurs with specific immune-engaged microenvironmental states and is associated with favorable outcomes after chemotherapy, echoing the survival advantage conferred by lymphocytic infiltration in aggressive subtypes [9] and situating SensReg within the broader framework of regulon-based tumor-microenvironment characterization we have developed for immunotherapy response [18, 19].

Several limitations temper these conclusions. The incremental analysis in ER-positive/HER2-negative disease rests on five cohorts with 6–13 pCR events each (2.0–4.3 events per variable), and its pooled estimate is correspondingly imprecise, with substantial heterogeneity (I^2^ = 75.7%) and a lower confidence limit close to zero; Firth penalisation was used for the per-cohort odds ratios to accommodate separation, but the GSE23988 estimate remains unstable. All analyses were retrospective and based on publicly available and single-institution data, with substantial between-cohort heterogeneity (I^2^ = 55% in the chemotherapy meta-analysis) reflecting differences in platform, regimen and pCR definition, and no cross-cohort batch correction was applied. Predictive value was concentrated in ER-positive disease and was modest in triple-negative and ER-negative, HER2-positive tumors, so SensReg is not a pan-subtype predictor. The institutional and spatial outcome analyses involved small numbers of events. The institutional recurrence-free survival association reached nominal significance but did so marginally (upper confidence limit 0.99), so these outcome findings are exploratory. Regulon activity is a model-dependent inference that depends on the chosen regulon resource and can be affected by tumor purity and composition, and the locked coefficients were applied without per-platform recalibration, which is a strength for reproducibility but may leave performance on the table for any individual assay. Finally, pCR is an imperfect surrogate at the individual-patient level, particularly in ER-positive, HER2-negative disease where its prognostic association is weaker, so clinical utility for treatment individualization cannot be assumed from pCR discrimination alone.

These limitations define a clear path to translation. Prospective evaluation, ideally embedded within neoadjuvant trials with adjudicated pCR, residual cancer burden and long-term outcomes, is required before clinical use, as is development and analytical validation of a core-needle-biopsy assay suitable for the pretreatment setting. Integration of SensReg with clinicopathological variables and with complementary immune markers, and testing in the context of chemoimmunotherapy and endocrine-based combinations, would clarify where it adds the most incremental value. If prospectively confirmed, a biologically interpretable, transferable and subtype-aware predictor such as SensReg could help identify ER-positive/HER2-negative tumors with differing likelihoods of neoadjuvant chemotherapy response and support prospective treatment-stratification studies.

## Methods

### Data sources and cohort assembly:

We assembled publicly available breast cancer transcriptomic datasets with treatment and response annotation from the Gene Expression Omnibus and other repositories, comprising five neoadjuvant chemotherapy (NAC) discovery cohorts (GSE194040, GSE23988, GSE25055, GSE25065, GSE28844), 35 independent chemotherapy-only NAC validation cohorts, 20 HER2-antibody treatment arms from 17 series, and 13 endocrine-therapy cohorts. Receptor status (ER, PR, HER2) and molecular subtype (IHC, and PAM50 where reported) were taken from the original studies, and chemotherapy regimens were grouped by mechanistic backbone as anthracycline, taxane, anthracycline–taxane, platinum-containing, or unspecified/other. The 40 chemotherapy-only NAC datasets comprised 3,852 patients after de-duplication (see below), and the 20 HER2-antibody arms, drawn from 17 series, comprised 739 patients of whom 734 were analyzable; receptor-subgroup analyses applied a single uniform rule to every panel, including a dataset’s subgroup only where it contributed at least 10 analyzable patients (non-missing score and response) with both outcome classes present, and reporting all subgroup sample sizes and pCR rates on that analyzable denominator. The unit of analysis throughout is the dataset × treatment arm, matching the forest plots in Fig. 3a,b: the 40 chemotherapy-only NAC datasets contribute one arm each, and the HER2-antibody set contributes 20 arms drawn from 17 series (GSE194040 contributing three arms and GSE123845 two). Chemotherapy subgroups comprised ER+/HER2 − n = 1,228 (22 strata), ER−/HER2 − n = 1,320 (25), ER+/HER2 + n = 195 (11) and ER−/HER2 + n = 246 (13); the trastuzumab subgroups comprised ER+/HER2 + n = 344 and ER−/HER2 + n = 320, each over 14 of the 20 arms, the remaining six per subgroup falling below the 10-patient threshold or contributing no patients of that receptor class. Subgroup AUROCs were compared by unpaired Mann–Whitney U test with Benjamini–Hochberg correction. Of the 3,852 chemotherapy-treated patients, 739 lacked complete ER/HER2 classification and therefore could not enter receptor-subgroup analyses. Among the 3,113 patients with complete receptor classification, a further 124 were excluded from the displayed receptor-subgroup analyses because their dataset×subgroup stratum contained fewer than 10 analyzable patients or lacked both response classes, leaving 2,989 patients across the four receptor-defined subgroups. Single-cell references for 80 cell types were obtained from the Human Protein Atlas (HPA) [20], and single-cell RNA sequencing data from 13 breast cancer patients from the Curated Cancer Cell Atlas (3CA)[21]. Bulk RNA-seq data for TCGA pan-cancer tumors were retrieved from the UCSC Xena browser[22] and GTEx healthy-tissue profiles from HPA. Accessions, sample sizes, treatment arms, clinical endpoints, and source publications for every chemotherapy and HER2-antibody dataset are listed in Fig. 3 and **Supplementary Table S3**; endocrine, spatial, single-cell and institutional cohort accessions are given with their respective analyses.

### Cohort de-duplication:

Because several public neoadjuvant series re-deposit the same tumors, the compendium was audited for patient-level redundancy before any performance estimate was computed. Every sample was keyed on its GEO sample accession (GSM) and, where available, on the submitter’s specimen label; labels were compared after normalizing case, whitespace and replicate suffixes (bis, -r1, rep1, dup). Labels that cannot discriminate between studies, namely bare integers, strings under four characters, and generic forms such as “Patient 3”, were excluded from cross-study matching, because identical generic labels recur across unrelated trials. Three classes of redundancy were resolved. First, GSE25066 was identified as the GEO SuperSeries of GSE25055 and GSE25065: all 444 of its samples were present in one or other SubSeries and none was unique, so GSE25066 was removed in its entirety and both SubSeries retained. Second, patient-level overlap was found among series sharing institution and platform, in which the same patients had been re-arrayed under different sample accessions; de-duplication gave precedence to the five discovery cohorts, which were left unaltered, and removed the redundant copies from the validation side, so that no validation patient contributed to model fitting. On this rule GSE20194 was reduced from 267 to 78 analyzable patients, GSE20271 from 178 to 131, and GSE22093 from 97 to 83; where a patient appeared in two validation cohorts the copy in the larger cohort was retained. Third, two series contained technical replicates, two arrays per patient: GSE28796 (explicit in the sample titles) and GSE22513 (every sample falling into one of fourteen pairs adjacent in accession number and identical in all recorded clinical fields). The first-deposited array of each pair was retained, reducing GSE28796 from 24 to 12 and GSE22513 from 28 to 14 patients. The final analysis set was verified to contain no sample accession and no discriminating specimen label occurring in more than one cohort, and no duplicate within any cohort.

### Data harmonization:

Raw expression data were processed with a per-platform handler pipeline: Affymetrix arrays were RMA-normalized from CEL files when raw data were available and otherwise rescaled to log2 series-matrix values, Illumina BeadArrays were quantile-normalized and log2-transformed, Agilent two-color arrays were retained on their native log2-ratio scale, and RNA-seq counts were normalized to log2(CPM + 1). Probe identifiers were mapped to current HGNC gene symbols through a four-source cascade (Bioconductor .db annotations, GEO GPL platform tables, ExpressionSet fData, and manual GPL-annotation fallback for platforms without a Bioconductor package), and duplicate symbols were collapsed by maximum-variance retention.

### Regulon activity inference.

Transcription-factor activities were inferred with VIPER on the DoRothEA [10] pan-cancer human regulon collection, applied independently within each dataset and identically to bulk, single-cell, and spatial data. VIPER estimates TF activity from the signed, coordinated expression of positively and negatively regulated target genes, yielding a functional readout of inferred protein activity rather than TF transcript abundance; positive scores indicate elevated activity and negative scores reduced activity. Only regulons with ≥ 10 target genes were retained. A common set of 1,158 regulons met the size criteria in all five discovery cohorts and entered the screen.

### Signature derivation and the SensReg score:

Within each discovery cohort, per-regulon discrimination of pCR was quantified by AUROC. Regulons were selected using three prespecified criteria: AUROC > 0.50 in all five discovery cohorts, mean AUROC ≥ 0.70, and Benjamini–Hochberg (BH) FDR < 0.01, yielding 13 regulons. Their z-scored activities were entered into a multivariable logistic regression, logit[P(pCR)] = β_0_ + Σ_i_₌_1…13_ β_i_·z_i_, fitted on the pooled discovery data. The intercept (β_0_ = −1.733) and 13 coefficients were frozen after discovery and were not re-estimated in any downstream dataset. The complete locked model coefficients are provided in **Supplementary Table S4**. Regulon activities were standardized within each validation cohort using the prespecified z-scoring procedure, after which the fixed coefficients were applied without outcome-based refitting to generate the continuous SensReg score (the logistic linear predictor). The primary endpoint was pathological complete response (pCR); where pCR was unavailable, the annotated binary clinical-response call was used; this applied to three of the 40 chemotherapy datasets: GSE143846 (n = 43), GSE29561 (n = 22) and GSE191127 (n = 20), 85 patients in total. SensReg is reported on the native scale of each data type: the standardized logistic linear predictor for bulk cohorts, per-cell regulon activity for single-cell data, and spot- or pseudobulk-level regulon activity for spatial data. Absolute values are therefore not comparable across modalities; within every analysis, “SensReg-high” is defined relative to that dataseťs own distribution. Two dichotomization rules were applied and are noted where used: for cell-level (single-cell) and spot-level (spatial) classification, a cell or spot was SensReg-high if its score met or exceeded the within-dataset median + MAD, else SensReg-low; the MAD scale constant was 1.4826 (the default consistency constant for a normal distribution) for single-cell data and 1 for spatial data; for patient-level survival grouping, patients were dichotomized at the within-cohort mean score into SensReg-high versus SensReg-low for Cox proportional-hazards analysis.

### Predictive performance, meta-analysis and receptor-defined subgroups.

Per-dataset AUROC with 95% DeLong confidence intervals was computed on the continuous score (pROC). For each dataset, the two-sided significance that AUROC differed from 0.5 was obtained from the Wilcoxon rank-sum comparison of scores between response groups, and these per-dataset p-values were Benjamini–Hochberg (BH) corrected across all datasets within an analysis set to yield the per-dataset FDR; between-group score differences were additionally tested by Welch's t-test. Per-cohort DeLong AUROC and standard error were combined by DerSimonian–Laird random-effects meta-analysis on the logit-AUROC scale and back-transformed, with heterogeneity summarized by I^2^; the arithmetic mean of dataset AUROCs and the pooled estimate were reported as distinct statistics. Cohorts with fewer than three samples in either outcome class, or a degenerate (zero) standard error arising from perfect class separation were excluded from pooling, as such cohorts contribute undefined or infinite inverse-variance weight; this excluded one dataset (GSE28796, one responder of 12), leaving 39 datasets (n = 3,840) in the chemotherapy-only meta-analysis; within the 35 independent validation datasets (n = 3,092), 34 datasets (n = 3,080) contributed to the validation-only pooled estimate. Global significance was assessed by a permutation null: at each of 2,000 iterations, outcome labels were shuffled within each cohort, per-cohort DeLong AUROC and standard error were recomputed, and the cohorts re-pooled by the identical DerSimonian–Laird procedure. The empirical p-value was (number of permuted pooled AUROCs ≥ observed + 1)/(2,000 + 1); with 2,000 iterations its smallest attainable value is 1/2,001 ≈ 5×10^−4^, so observed pooled AUROCs exceeding all permuted values are reported as p < 5×10^−4^. The permutation null was not converted to a normal-tail p-value, as such an approximation is unreliable in the far tail of a 2,000-draw null. The locked score was applied without refitting to the HER2-antibody and endocrine cohorts. For survival analysis, patients were dichotomized at the within-cohort mean score (high versus low), and association with the cohorťs reported endpoint (overall, recurrence-free, or metastasis-free survival) was estimated by Cox proportional-hazards regression as hazard ratios with 95% confidence intervals.

Chemotherapy performance was further evaluated within ER+/HER2−, ER−/HER2−, ER+/HER2 + and ER−/HER2 + groups; a dataset-subgroup stratum was included only with subgroup N ≥ 10, and subgroup means were unweighted arithmetic means of eligible strata. Pairwise differences among the four groups used the unpaired Mann–Whitney U test with BH correction across comparisons, and global differences used a permutation test. Subgroup pCR rates were reported descriptively as strict pCR = 1 counts over the corresponding subgroup denominators across eligible strata. Inclusion rules differ between analyses according to what each estimator requires, and are stated together here. Dataset-level AUROC analyses required at least three patients in each outcome class, which excludes GSE28796 alone. Receptor-subgroup and ER+/HER2 − analyses required at least ten analyzable patients with both outcome classes present. The age-and-grade model required complete age, grade, score and response. In the untreated TCGA-BRCA subset (n = 292 primary tumors with no recorded neoadjuvant or adjuvant systemic therapy), SensReg was tested for prognostic association by Cox regression for overall survival and progression-free interval, with Kaplan–Meier visualization and time-dependent AUROC. The hazard ratios reported in the Results are for SensReg-high versus SensReg-low tumors, dichotomized at the cohort mean.

### Research-based recurrence score.

A research approximation of the 21-gene recurrence score (RS) was computed per sample from the Oncotype DX genes following the Paik et al. structure (GRB7, ER, proliferation and invasion group scores with published reference floors and coefficients, rescaled to 0–100) [23], via genefu::oncotypedx [24] where available, an equivalent base-R implementation otherwise, and a reference-gene-normalized variant as fallback. This is a research approximation, not the commercial assay.

### Recurrence-score-intermediate analysis.

A dataset was analyzed if it had ≥ 10 ER+/HER2 − chemotherapy samples with a pCR label (19 cohorts, n = 910, 118 pCR; the remaining ER+/HER2 − cohorts lacked a computable RS and entered only score-independent analyses). Within each cohort, ER+/HER2 − samples were assigned to low/intermediate/high RS strata by two definitions: (i) TAILORx-calibrated, quantile-mapping each cohorťs RS to a reference distribution and cutting at the TAILORx randomization boundaries, which are a trial randomization range rather than a guideline-defined intermediate category (low < 11, intermediate 11–25, high ≥ 26) [13], with absolute boundaries approximate because the reference is illustrative; and (ii) cohort median ± MAD (R mad, constant 1.4826; low < median − MAD, high > median + MAD, else intermediate), with quartile (25/50/25) splits as robustness checks. SensReg discrimination for pCR within the intermediate stratum was estimated per cohort (DeLong AUROC) and pooled at patient level (mixed-effects OR per 1 SD and a single pooled DeLong AUROC), with a DerSimonian–Laird random-effects meta-analysis on the logit-AUROC scale as sensitivity analysis.

### Robustness and null-signature analyses

Redundancy among the 13 regulons was characterized by pairwise regulon-activity correlation and target-gene overlap. Dependence on individual components was assessed by leave-one-regulon-out recomputation of the mean discovery AUROC. Specificity was assessed in the 35 held-out validation cohorts rather than in the discovery cohorts. Because the 13 regulons were selected on mean discovery AUROC ≥ 0.70 and no non-selected regulon reached that threshold, a discovery-cohort comparison against non-selected regulons is fixed by the selection rule and cannot test it. SensReg was scored not as its fitted logistic model but as an unfitted equal-weight composite, summarized by the mean per-regulon AUROC of its 13 members across the validation cohorts, and compared with 10,000 random 13-regulon sets summarized identically, drawn either from all 1,145 non-selected regulons or from the 118 sensitivity-matched non-selected regulons whose discovery mean AUROC was 0.60–0.70. Pool membership was defined on discovery in both cases, so the comparison asks whether the discovery ranking generalizes. Both SensReg and the random sets are summarized the same way, with no coefficient fitting on either side. The empirical P value was (number of random sets with statistic ≥ SensReg + 1)/(number of permutations + 1).

### Reference signatures, orthogonality and functional characterization

SensReg was compared with 17 reference signatures scored by rank-based singscore without imputing missing genes; gene sets are provided in Supplementary Table S5. Head-to-head analyses were restricted to the 35 independent validation cohorts; GSE28796 failed the prespecified ≥ 3 responders and ≥ 3 non-responders criterion, leaving 34 evaluable cohorts. Comparator direction was fixed from mean AUROC across the five discovery cohorts before validation and was never selected from validation outcomes. Per-cohort AUROCs were compared by exact two-sided paired Wilcoxon signed-rank tests with BH correction across 17 comparators. Incremental value was ΔAUROC = AUROC(z-comparator + z-SensReg) − AUROC(z-comparator), with both scores standardized within cohort and summed equally without outcome-derived weights. Per-cohort increments were averaged and tested against zero by exact two-sided one-sample Wilcoxon signed-rank tests with BH correction across 17 comparisons. Independent association was assessed in cohort-specific models containing SensReg and all 17 comparators simultaneously, each standardized to 1 SD. To stabilize estimation of 18 correlated predictors, coefficients were ridge-penalized with the intercept unpenalized, maximizing log-likelihood − (λ/2)Σβ^2^ with prespecified λ = 1, equivalent to independent N(0,1) priors on per-SD log-ORs and not outcome-tuned. Models used damped Newton-Raphson with step halving to maximum |Δβ|<10^−10^; covariance was estimated by the ridge sandwich (*X*′*WX* + λ*D*)^−1^(*X*′*WX*)(*X*′*WX* + λ*D*)^−1^. Cohorts required ≥ 3 patients per outcome class, no zero-variance predictors, full rank and convergence; 27/34 qualified. Dataset-specific log-ORs were pooled by REML random-effects meta-analysis with BH correction across 18 predictors. Because the median was 1.2 pCR events per predictor and collinearity remained high (VIF up to 38), this analysis is exploratory; ridge ORs are shrinkage-biased toward 1, their CIs are conditional on λ, and comparator coefficients should not be interpreted as stable effect sizes.

Hallmark over-representation of SensReg-associated up-regulated targets was tested against the 50 MSigDB Hallmark sets (h.all.v2022.1.Hs) [25] by the hypergeometric test with BH-adjusted q values. Redundancy among enriched pathways was resolved by Concept Signature Enrichment Analysis (CSEA2, IndepthPathway) [26] against the combined Hallmark and canonical-pathway collections (c2.cp.v2022.1.Hs): concept similarity used the Ochiai coefficient (minimum overlap 10 genes), uniConSig via cal.uniConSig (rm.overfit = FALSE); the top 50 pathways at nominal P < 0.05 were disambiguated at Bonferroni P < 0.01, and the top 15 non-redundant pathways were connected by pathwayAssociation.

Orthogonality to proliferation was assessed in TCGA-BRCA by Spearman correlation of SensReg with Hallmark pathway scores and by partial Spearman correlation adjusting separately for MKI67 and a Cell Cycle score. Partial Spearman was computed by rank-transforming variables, residualizing ranked SensReg and ranked pathway on the ranked covariate by linear regression, and taking the Pearson correlation of residuals (P from the t statistic), with BH correction. Pairwise relationships among SensReg and the reference signatures were summarized by a hierarchically clustered Spearman correlation matrix. Signature-panel structure was further examined by principal component analysis of the per-sample score matrix (prcomp, centered and unit-scaled) in 1,104 TCGA-BRCA tumors, with variable loadings on the first two components colored by contribution. Loadings on all retained components were inspected, not only the two that are plotted, because a signature may be separated from a comparator block on a lower-variance axis while appearing close to it on the first two. All TCGA-BRCA analyses draw on the same 1,104 tumors with a SensReg score, and each panel uses the complete-case subset for the variable concerned: 1,054 with a PAM50 subtype call (Fig. 7a), 949 with a deep-learning TIL percentage (Fig. 7b), 1,065 with a purity estimate (Fig. 7c) and all 1,104 for CIBERSORT (Fig. 7d); no tumor is excluded for any other reason. TCGA-BRCA associations shown in Fig. 7 used the following sources. PAM50 intrinsic subtype [27], was taken from the subtype annotation distributed with the TCGA-BRCA data, and tumors were classified SensReg-positive or SensReg-negative at the cohort mean of the SensReg score. Tumor-infiltrating lymphocyte fraction was the deep-learning-derived TIL percentage of Saltz et al. [28], computed from the TCGA diagnostic H&E images. Tumor purity was taken from the pan-cancer tumor-microenvironment resource of Bagaev et al.[29]. Immune composition was estimated by CIBERSORT [30] in absolute mode (“cibersort_abs”) with the LM22 signature matrix, run through the immunedeconv wrapper [31]. Associations between the SensReg score and each of these variables were quantified by two-sided Spearman correlation, with Benjamini–Hochberg correction across the 22 LM22 cell types.

### Clinical utility.

Clinical utility was assessed primarily in ER+/HER2 − disease, the subgroup in which the decision to administer neoadjuvant chemotherapy is not dictated by receptor status, restricting to cohorts with at least 10 ER+/HER2 − patients; ER−/HER2−, ER−/HER2 + and ER+/HER2 + chemotherapy-treated subgroups were assessed separately. Per-cohort AUROCs of the continuous SensReg score were pooled by DerSimonian–Laird random-effects meta-analysis, and decision-curve analysis estimated net benefit across threshold probabilities from cohort-adjusted, cross-validated predicted probabilities. This step is an exception to the locked-model framing used elsewhere: decision-curve analysis requires predictions on the probability scale, and the locked linear predictor is not itself calibrated to the pCR probability of any given cohort. Probabilities were therefore obtained by fitting a cohort-adjusted logistic model to the locked score and taking out-of-fold predictions under repeated stratified cross-validation. The regulon coefficients themselves were not re-estimated at any point. Net benefit at threshold t was the true-positive proportion minus the false-positive proportion weighted by t/(1 − t), compared with treat-all and treat-none strategies; the treat-all reference crosses zero net benefit at the subgroup response prevalence.

### Incremental value of SensReg beyond clinicopathological (age and grade) variables.

Analysis was restricted to ER+/HER2 − tumors with complete Age, Grade, pCR and SensReg data, yielding 390 patients (44 pCR) across five cohorts. A clinical logistic model (age + grade) was compared with a clinical-plus-SensReg model (age + grade + SensReg score), both L2-penalized (scikit-learn LogisticRegression, C = 1.0, lbfgs solver, max_iter = 2000), by ten-times-repeated three-fold stratified cross-validation (StratifiedKFold, shuffle=True, seeds 1000–1009 shared by both models) with predictors standardized on training-fold parameters and held-out probabilities averaged across repeats; per-cohort incremental discrimination ΔAUROC = AUROC(age+grade+SensReg) − AUROC(age+grade) was computed on the averaged out-of-fold probabilities, with 95% CIs and standard errors from a 2,000-replicate paired patient-level bootstrap (numpy default_rng seed 20260825). In addition, a single multivariable logistic model per cohort (pCR ~ SensReg + age + grade, SensReg and age standardized within cohort, grade ordinal) was fitted by Firth's penalized likelihood to accommodate separation, and the adjusted odds ratio for pCR per 1-SD SensReg is reported with a Wald 95% CI. In both analyses the SensReg regulon coefficients were fixed (never re-estimated), and dataset-level estimates (ΔAUROC and log-odds ratios) were pooled by REML random-effects meta-analysis with a two-sided normal-approximation p value and heterogeneity summarized by I^2^.

### Single-cell analysis:

Breast cancer single-cell RNA-seq data from 13 patients (18,885 cells across three series) were scored using the SensReg framework. Analyses focused on the malignant compartment and seven major non-malignant compartments: T cells, macrophages, B cells, fibroblasts, endothelial cells, NK cells and dendritic cells. A cell was classified as SensReg-high if its activity met or exceeded the within-dataset median + MAD (scale constant = 1.4826). For each patient, the SensReg-high cell ratio for a compartment was defined as the number of SensReg-high cells in that compartment divided by the total number of cells in that patient’s sample; thus, this ratio reflects the contribution of each compartment to the sample-wide SensReg-high population rather than the within-compartment frequency of SensReg-high cells. Both measures are reported in the Results. For each patient, the SensReg-high cell ratio in each non-malignant compartment was compared with that in the malignant compartment using a two-sided paired Wilcoxon signed-rank test, with Benjamini–Hochberg correction across comparisons.

### Spatial transcriptomic analysis

The BCTL TNBC cohort comprised 94 tumors (83 chemotherapy-treated). Spot-level SensReg activity was mapped to histological coordinates. Enrichment of SensReg-high spots across annotated histological compartments and across 14 predefined spatial megaclusters was quantified by odds ratios with BH-FDR. Because spots are nested within slides and patients, odds ratios computed over pooled spots treat correlated observations as independent and yield anticonservative p values; enrichment was therefore also estimated within each patient and pooled across patients by DerSimonian–Laird random effects, and the patient-pooled estimates with their between-patient heterogeneity are the ones reported. Sample-level spatial pseudo-bulk scores were used for the tumor-immune microenvironment categories and the 14 spatial megaclusters defined for this cohort [15], and for Bareche TNBC subtype assignments [32] (global, tumor, stroma). Among chemotherapy-treated tumors with evaluable outcome, survival by spatial SensReg status was assessed by Kaplan–Meier analysis with Cox proportional-hazards models for overall and distant recurrence-free survival; hazard ratios are reported with profile-likelihood 95% confidence intervals and likelihood-ratio p values, the same basis used for the institutional cohort, because the event counts are small. Spatial SensReg was the locked score computed on the pseudobulk count matrix and dichotomized at the cohort mean. For the necrosis-distance analysis, each spot on a slide carrying annotated necrosis was assigned the Euclidean distance to the nearest necrotic spot and binned; distances are reported in spot pitches, one pitch being the nearest-neighbour spot spacing of the array (19.87 image pixels, coefficient of variation 0.29%). The gradient was summarized pooled across spots by Spearman correlation and Kruskal–Wallis test across bins, and again within each patient by Spearman correlation with Benjamini–Hochberg correction; because pooled-spot statistics treat spots nested in the same tumor as independent, the per-patient correlations and a one-sample Wilcoxon test of those correlations against zero are the inferential result.

### Institutional validation in the UPMC TNBC cohort:

Institutional validation was restricted to the 48 chemotherapy-only patients (from 67 patients with a SensReg score; those receiving concurrent targeted or endocrine therapy, endocrine therapy alone, or with missing treatment type were excluded) from our previously reported UPMC TNBC cohort (GSE312235); 44 of the 48 had evaluable overall-survival follow-up and 45 had evaluable recurrence-free-survival follow-up. Median follow-up was 62 months (5.2 years; range 7–169) for overall survival and 65 months (5.4 years; range 0–169) for recurrence-free survival. Continuous SensReg scores were compared between patients with and without recorded recurrence during available follow-up using a one-tailed Mann–Whitney U test, with the direction specified a priori from the chemotherapy-sensitivity hypothesis; recurrence frequencies were additionally summarized in SensReg-low and SensReg-high groups defined by the cohort mean. These two comparisons used all 48 patients and are reported as descriptive summaries of observed recurrence: they take no account of differing follow-up duration, so patients recorded as free of recurrence include those observed for less than 10 years, and they are not offered as time-to-event inference. The censoring-aware comparison is the recurrence-free survival analysis. The overall-survival and time-dependent ROC analyses included 44 patients with evaluable OS follow-up, whereas recurrence-free survival analysis included 45 patients with evaluable RFS follow-up. Time-dependent overall-survival discrimination at 2, 5 and 10 years was evaluated using cumulative/dynamic ROC analysis with inverse-probability-of-censoring weighting and Kaplan–Meier estimation of the censoring distribution. The ROC orientation was fixed a priori from the same chemotherapy-sensitivity hypothesis that specified the one-tailed Mann–Whitney direction: higher SensReg was hypothesized to indicate chemosensitivity and therefore lower mortality risk, so the score was multiplied by − 1 solely for ROC calculation to give a risk-oriented marker that increases with mortality risk. The orientation was not selected from the observed direction in these data. Reported AUC values therefore quantify discrimination, whereas the biological direction remains that higher SensReg indicates more favorable survival.

## Supplementary Material

Supplementary Files

This is a list of supplementary files associated with this preprint. Click to download.

• SupplementaryTables.xlsx

• SensRegChemoSupplementaryFigure.pdf

## Figures and Tables

**Figure 1:**
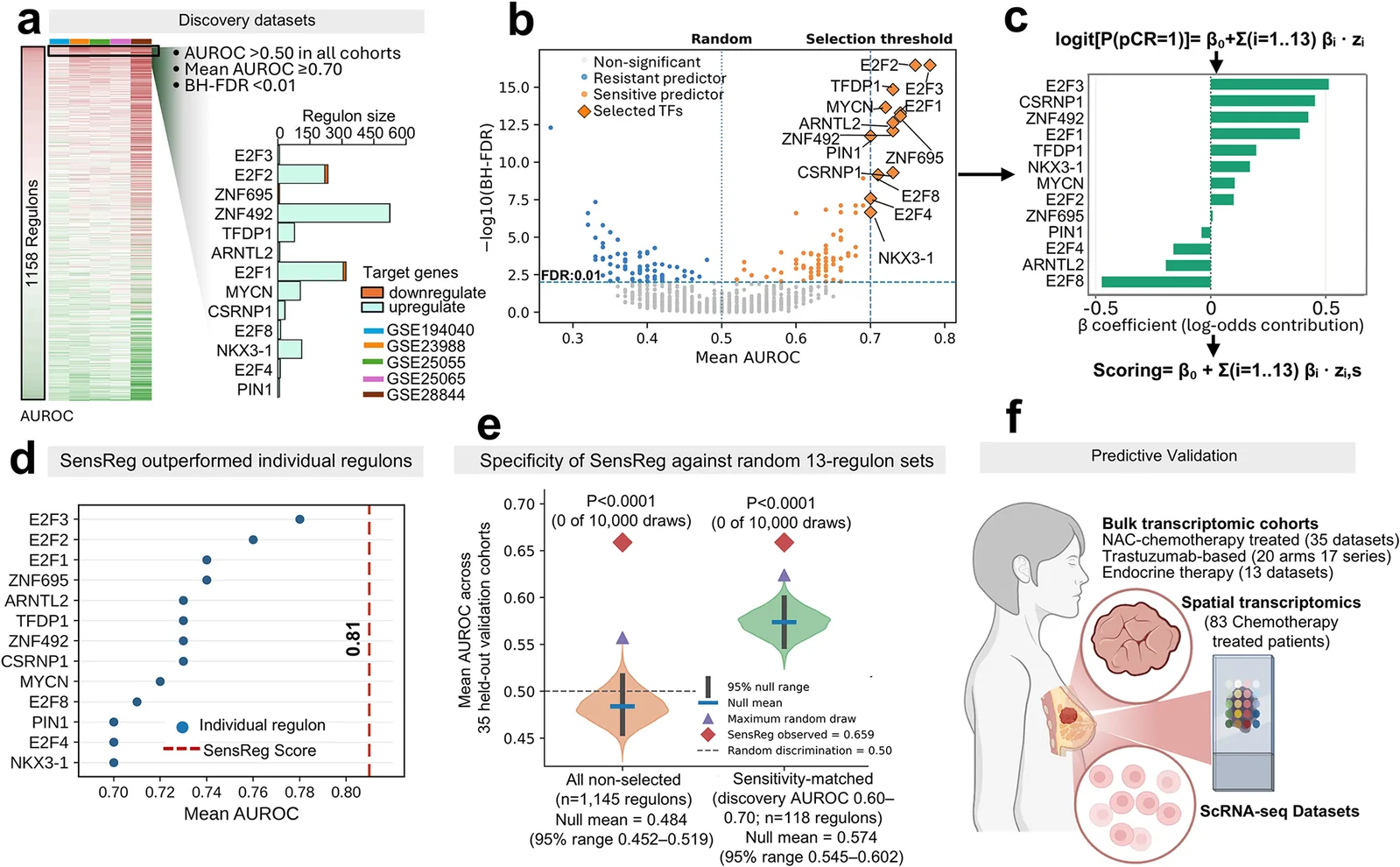
Derivation of the SensReg transcription-factor signature for neoadjuvant chemotherapy response, and its specificity in held-out cohorts. (**a**) Regulon activity was inferred for 1,158 regulons across five NAC discovery cohorts (GSE194040, GSE23988, GSE25055, GSE25065, GSE28844) and ranked by per-cohort AUROC for pCR. Thirteen regulons met all prespecified criteria (AUROC > 0.50 in every cohort, mean AUROC ≥ 0.70, BH-FDR < 0.01); bars show target-gene count, colored by direction of regulation. (**b**) Volcano plot of mean AUROC vs −log10(BH-FDR); the 13 selected regulons (diamonds) exceed both the mean-AUROC and FDR = 0.01 thresholds. (**c**) The 13 z-scored activities were combined in a multivariable logistic model, logit[P(pCR=1)] = β0 + Σi=1..13 βi·zi, fitted on discovery cohorts. Bars show each β coefficient (**d**) Mean per-regulon AUROC across the five discovery cohorts vs the composite SensReg score (dashed line, 0.81), which exceeds every constituent. (**e**) Specificity in 35 held-out cohorts. SensReg, as an unfitted equal-weight composite (mean per-regulon AUROC 0.659), was compared with 10,000 identically summarized random 13-regulon sets from all 1,145 non-selected regulons (null mean 0.484, 95% range 0.452–0.519, max 0.557) or 118 sensitivity-matched non-selected regulons (discovery AUROC 0.60–0.70; null mean 0.574, 95% range 0.545–0.602, max 0.624). No random set reached the observed value (empirical P < 0.0001, 0/10,000). Violins, null distributions; bar, 95% null range; dashed line, random discrimination (0.50). (**f**) Validation strategy: bulk cohorts (chemotherapy-only NAC, 35; trastuzumab-based, 20; endocrine, 13), spatial transcriptomics (chemotherapy-treated slides), and scRNA-seq datasets.

**Figure 2:**
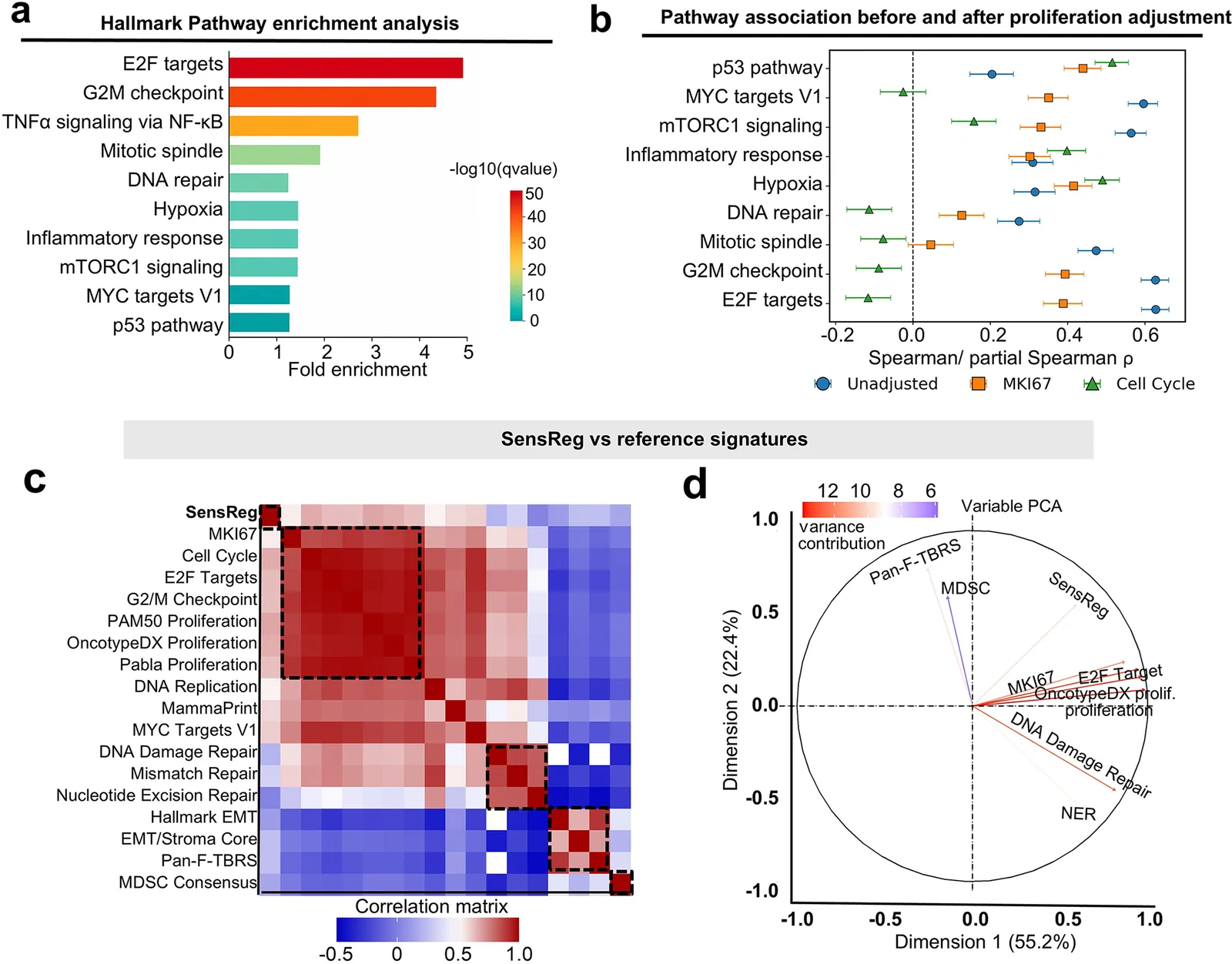
SensReg captures a proliferation-linked program distinct from the proliferation signatures evaluated here. (a) Hallmark over-representation of the SensReg-associated up-regulated target genes (see Methods) against all 50 MSigDB Hallmark sets by hypergeometric test; the 10 terms shown are illustrative, not the complete result. Bars, fold enrichment; color, −log10(q-value); E2F targets and G2M checkpoint are most enriched. Because six of the 13 regulons are E2F-family members or TFDP1, this is expected by construction, a positive control. (b) SensReg versus cancer-hallmark pathway scores as Spearman ρ, unadjusted and partial (adjusting separately for MKI67 or a multi-gene cell-cycle score); points and whiskers, ρ and 95% CI. The cell-cycle-score adjustment collapsed canonical cell-cycle associations negative, whereas MKI67 adjustment only attenuated them; hypoxia and inflammatory-response programs were retained under both and the p53-pathway association strengthened. (c) Pairwise correlation matrix of SensReg with the 17 reference variables; dashed boxes, hierarchical-clustering blocks. SensReg falls within the broad proliferation block but is its weakest member (ρ 0.53–0.67 versus 0.91–0.99 among the other signatures), correlates with MKI67 at only ρ = 0.54, is unrelated to nucleotide excision repair (ρ = 0.02, p = 0.51), and is the only member positively correlated with EMT, stromal and myeloid programs. (d) Variable PCA (Dimension 1, 55.2%; Dimension 2, 22.4%; n = 1,104 TCGA-BRCA tumors); arrow length and color, each variable's contribution. SensReg carries the smallest Dimension-1 loading of any proliferation-associated variable. On Dimension 5 (4.1% of variance) it loads +0.69 while six of eight comparators load negatively, including MKI67 (−0.32) and Pan-F-TBRS (−0.60).

**Figure 3:**
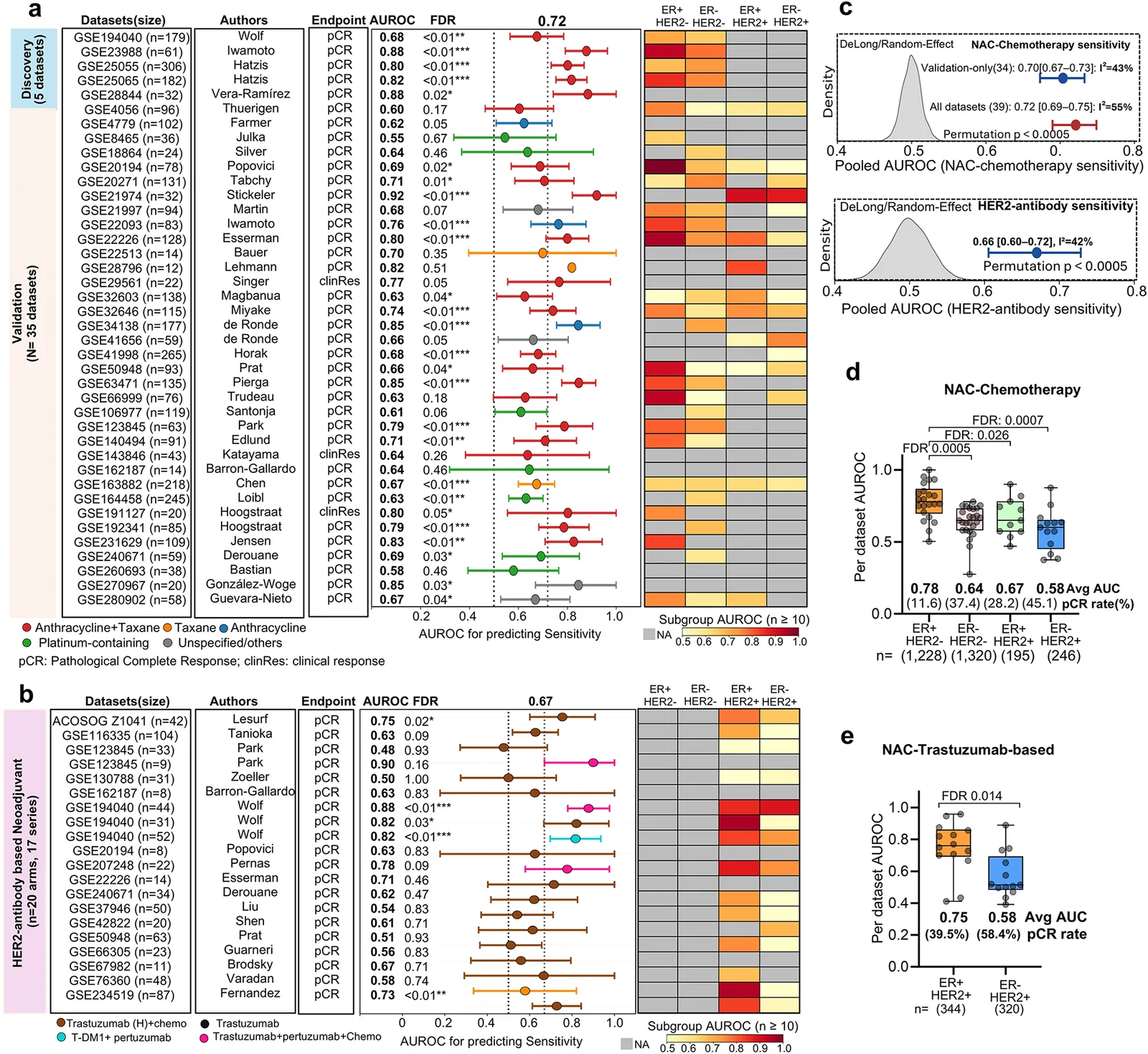
SensReg predicts neoadjuvant treatment sensitivity across 40 chemotherapy and 20 HER2-antibody arms (17 series), with predictive value concentrated in ER-positive disease. (a) AUROC (point) with 95% CI (whiskers) for SensReg predicting pCR or clinical response; five discovery and 35 validation chemotherapy-only NAC cohorts; color, regimen backbone regrouped by agents administered (inclusive grouping; group definitions, per-group n and per-cohort regimens, Supplementary Table S3). Right heatmap, receptor-subtype composition per cohort. GSE28796 (n = 12, one responder), no CI, not pooled (pre-specified exclusion: fewer than three samples in an outcome class). (b) Forest plot, the 20 HER2-antibody neoadjuvant arms (n = 734). Three arms (n = 127) are randomised HER2-directed arms of discovery series GSE194040 (I-SPY2): P + trastuzumab + pertuzumab (44), P + trastuzumab (31), T-DM1 + pertuzumab (52); distinct from the paclitaxel-only discovery arm (n = 179, no HER2-directed drug), with no patients shared with derivation, so the caveat is shared trial and platform only. (c) DeLong/random-effects pooling with a 2,000-iteration permutation null (empirical p resolution 5×10^−4^) for chemotherapy-only NAC (39 of 40 datasets poolable, n = 3,840), validation-only (34 of 35, n = 3,080) and HER2-antibody (20 arms, n = 734) sets; pooled AUROCs, 95% CIs and I^2^ as reported in Results. Each observed pooled AUROC exceeded all 2,000 permuted values (p < 5×10^−4^; chemotherapy-only ≈ 20 null SDs above the null mean). Gray histograms, permutation nulls. (d) Per-dataset AUROC by receptor subtype (chemotherapy-only NAC): ER+/HER2− 0.78 (n = 1,228), ER−/HER2− 0.64 (n = 1,320), ER+/HER2+ 0.67 (n = 195), ER−/HER2+ 0.58 (n = 246); sample size and pCR rate annotated, pairwise BH-FDR shown. (**e**) By subtype (trastuzumab-based): ER+/HER2+ 0.75 vs ER−/HER2+ 0.58 (BH-FDR 0.014, unpaired Mann–Whitney U; n = 344 and 320,14 arm-level strata each).

**Figure 4:**
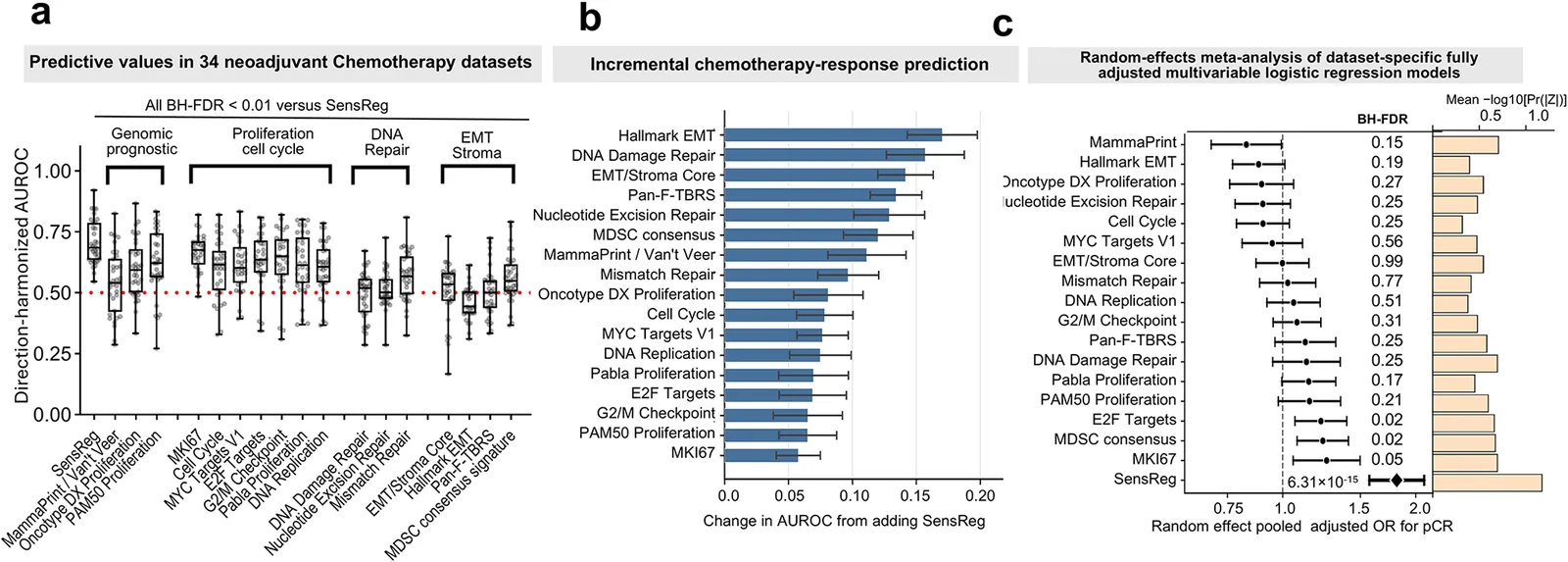
SensReg outperforms established prognostic and proliferation signatures and adds independent predictive value in multivariable models. (a) Per-cohort AUROC for response across the 34 evaluable validation cohorts against 17 reference signatures, grouped by class. Boxes, median and IQR; dashed line, random (0.50). Cohorts with fewer than three responders or non-responders (GSE28796, n = 12) and the five discovery cohorts are excluded. Orientation was fixed once from each signature's mean discovery-cohort AUROC, so points below 0.50 mark direction reversal, not poor discrimination; only DNA Damage Repair was reoriented. Pairwise comparisons used two-sided paired Wilcoxon signed-rank tests on the per-cohort AUROCs, with exact p and BH correction across the 17 comparators. (b) ΔAUROC when SensReg is added to each signature, ranked. Both scores were standardized to unit SD within cohort and summed with equal weight, so the combination uses no outcome information. Bars, mean ΔAUROC; whiskers, 95% CI. Increments were tested against zero by two-sided one-sample Wilcoxon signed-rank with BH correction; none exceeded SensReg alone. (c) Cohort-specific logistic models of SensReg plus all 17 comparators, standardized to 1 SD within cohort, ridge-penalized (see Methods) and pooled by REML random-effects meta-analysis with BH correction across 18 predictors. Forest, pooled adjusted odds ratio per 1 SD with 95% CI; bars, mean −log10 p; column, BH-FDR; ordered by odds ratio. Estimable in 27 of 34 cohorts; the seven exclusions were two too small and five lacking complete measured comparator. At a median of 1.2 pCR events per predictor and with comparators collinear, panel c is exploratory and its odds ratios are not effect sizes.

**Figure 5:**
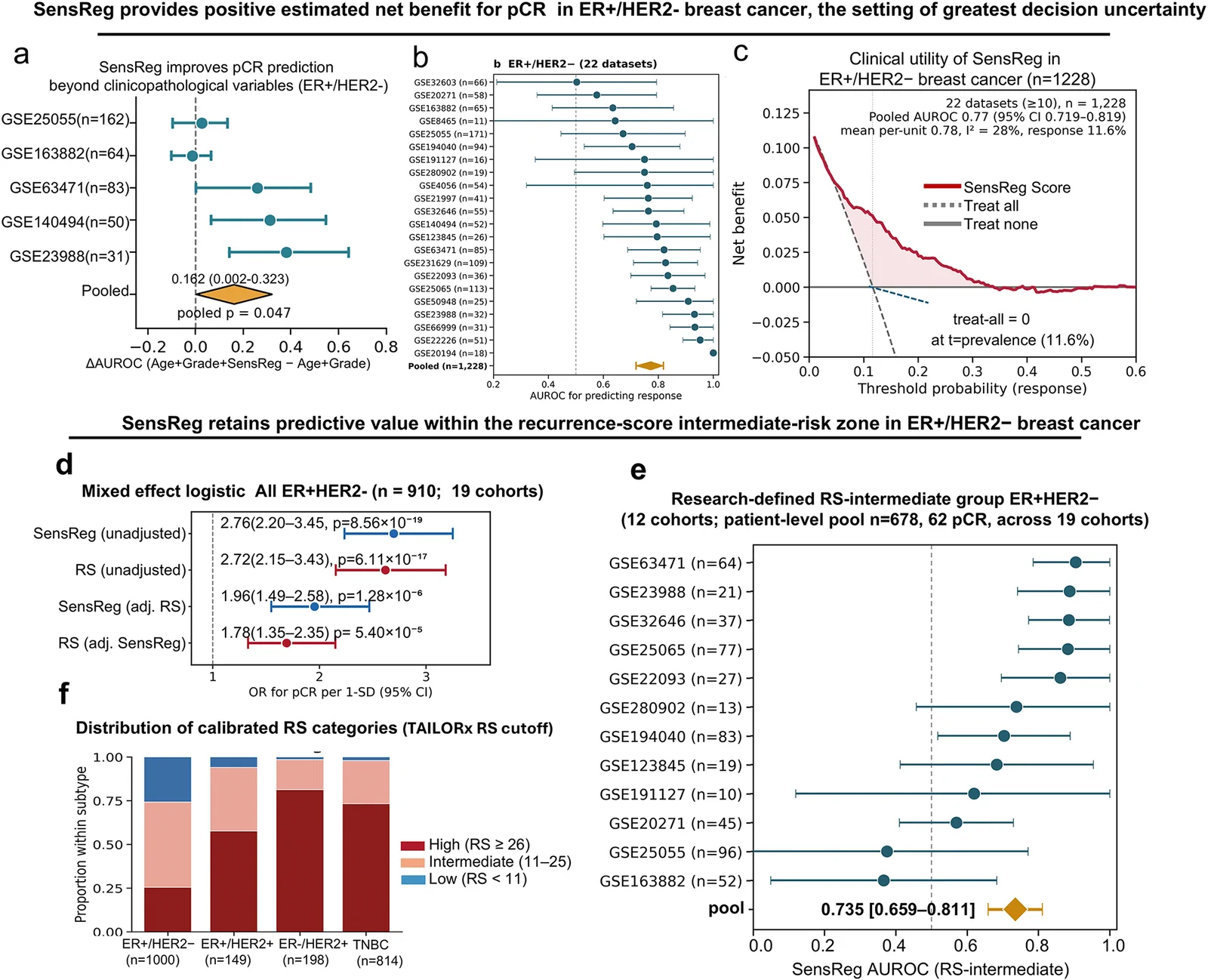
SensReg improves pCR prediction, provides clinical net benefit, and adds information beyond a research recurrence score in ER-positive/HER2-negative breast cancer. (a) ΔAUROC from adding SensReg to an age-and-grade model across the five ER+/HER2− cohorts with complete age, grade, score and pCR (GSE23988, GSE140494, GSE25055, GSE63471, GSE163882), with the random-effects pooled estimate; points and whiskers, ΔAUROC and 95% CI. Two are discovery cohorts, so this panel is not independent of derivation. (b) Per-cohort AUROC with 95% CI across 22 ER+/HER2− cohorts and the pooled estimate; four are discovery cohorts; restricting to the 18 validation cohorts leaves it unchanged. (c) Decision-curve analysis in the pooled ER+/HER2− population (n = 1,228; pCR prevalence 11.6%), using out-of-fold probabilities from a cohort-adjusted logistic model of the locked score, against treat-all and treat-none. (d) Mixed-effects logistic regression across 19 ER+/HER2− cohorts with computable recurrence scores (n = 910; 118 pCR): odds ratios per 1 SD for SensReg and RS, univariable and mutually adjusted. (e) SensReg discrimination within the RS-intermediate stratum, defined a priori as the cohort median ± MAD (constant 1.4826; pool n = 678, 62 pCR, 19 cohorts). Points and whiskers, cohort-specific AUROCs for the 12 with an estimable value; diamond, the patient-level pooled estimate. Two run in reverse (GSE25055, GSE163882). Under the pre-specified minimum-event rule (five patients per outcome class) the five qualifying cohorts give 0.743; the quartile and TAILORx-calibrated robustness definitions give 0.722 and 0.700. (f) Calibrated RS categories by receptor subgroup using TAILORx-aligned thresholds (low <11, intermediate 11–25, high ≥26); the RS is a research approximation, not the commercial assay.

**Figure 6:**
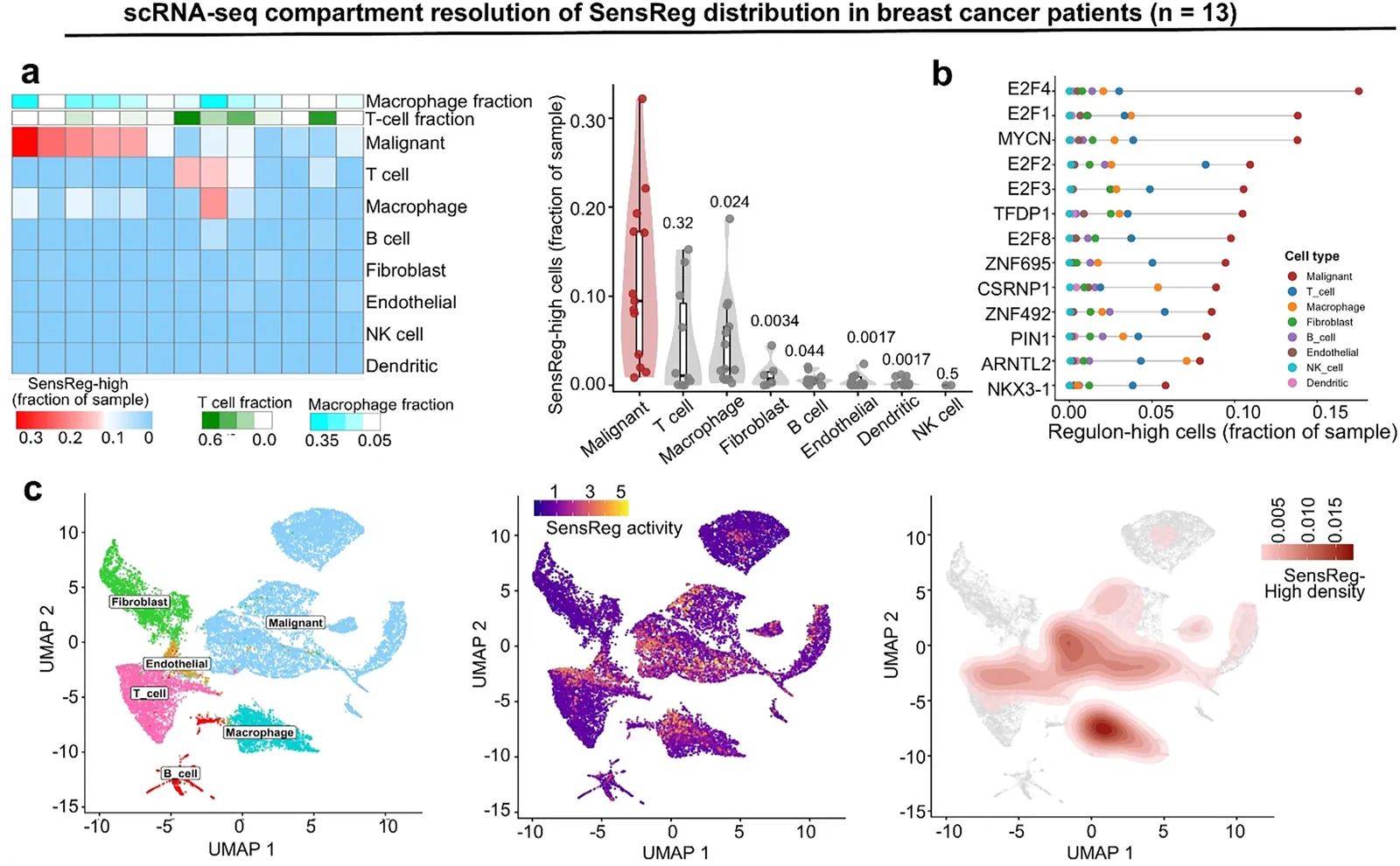
Single-cell compartment resolution of SensReg-high cells. (a) scRNA-seq of 13 breast cancer patients. Left heatmap, SensReg-high cell ratio per compartment with per-sample macrophage and T-cell fractions; right, the same ratio by compartment. The ratio is SensReg-high cells in a compartment over all cells in that patienťs sample, so it reflects compartment abundance as well as per-cell frequency; SensReg-high is activity above median + MAD (scale constant 1.4826), defined per dataset. Per-cell SensReg-high frequencies were 18.7% (malignant), 36.8% (macrophage), 31.7% (endothelial), 24.3% (T cell), 14.6% (B cell) and 3.8% (fibroblast). The malignant compartment had the highest ratio, exceeding dendritic, endothelial, fibroblast, macrophage and B cell (paired Wilcoxon signed-rank versus malignant, BH-corrected across seven comparisons, q < 0.05 each). T cell and NK cell ran in the same direction but were non-significant; at n = 2 patients the smallest attainable two-sided exact signed-rank p is 0.5, so the NK comparison is uninformative rather than negative. (b) Per-regulon activation by cell type for the 13 SensReg regulons, on the panel-a denominator, so values run 0–0.18 rather than 0–100%; activity concentrates in malignant cells across E2F-family and associated regulons. (c) UMAP embeddings (13 patients pooled): compartments (left), per-cell SensReg activity (middle), SensReg-high cell density (right). Kernel density on a UMAP is sensitive to embedding tightness: macrophages occupy 97.1 cells per unit area against 44.0 for malignant cells, so this panel shows where high-activity cells lie, not compartment preference.

**Figure 7:**
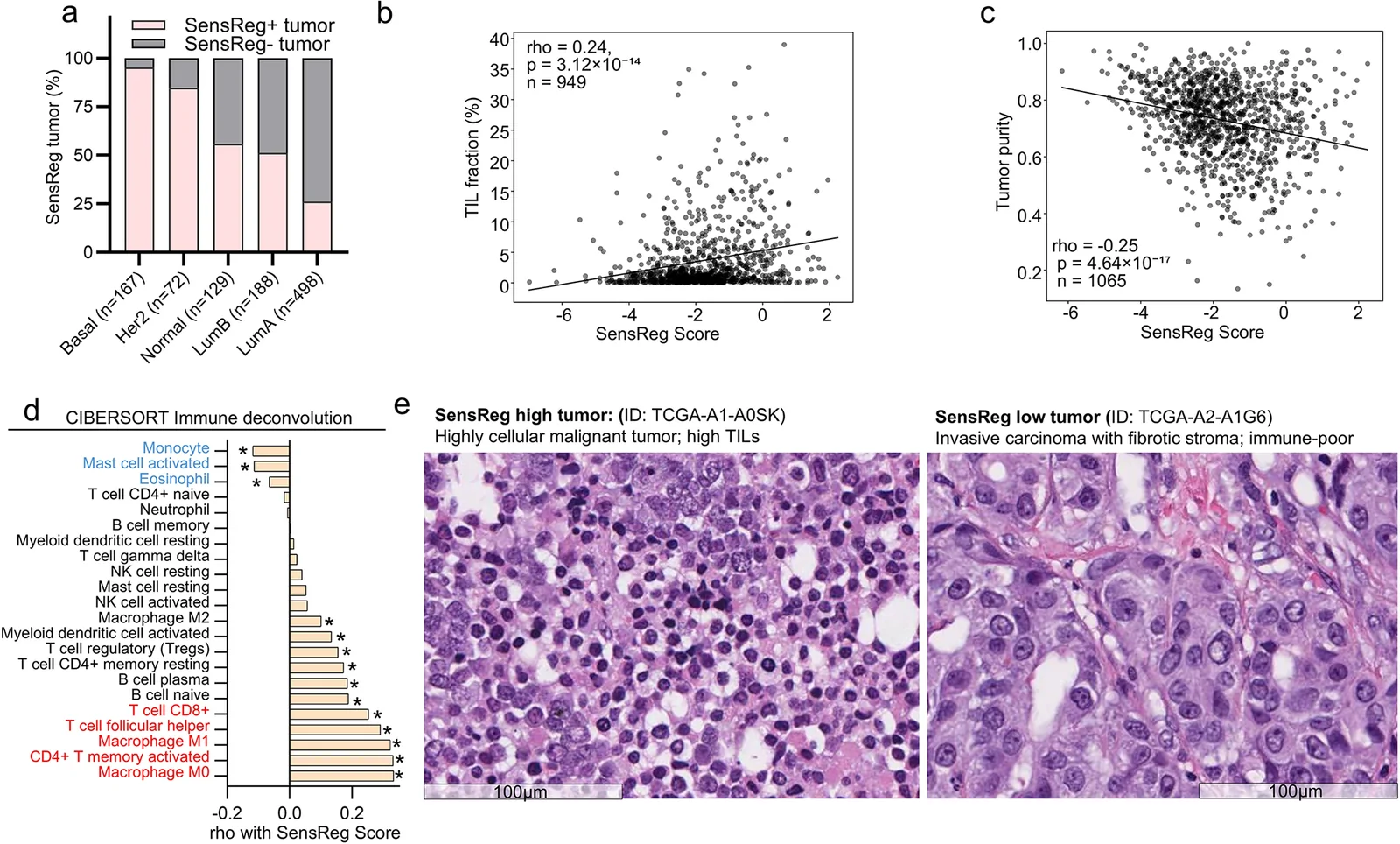
In TCGA-BRCA, SensReg-high tumors are enriched in proliferative subtypes and associate modestly with higher immune infiltration and lower tumor purity. All panels draw on 1,104 tumors with a SensReg score; each uses the complete-case subset for its variable. (a) Proportion of SensReg-positive versus SensReg-negative tumors by PAM50 subtype (n = 1,054), classified at the cohort mean of the score. (b) SensReg score versus deep-learning tumor-infiltrating lymphocyte fraction (n = 949). (c) SensReg score versus tumor purity (n = 1,065). Panels b and c report two-sided Spearman correlations. (d) CIBERSORT LM22 immune deconvolution in absolute mode (n = 1,104): bars, two-sided Spearman ρ between each abundance estimate and the SensReg score, with Benjamini-Hochberg correction across the 22 cell types; asterisks mark FDR < 0.05, and red and blue labels the strongest positive and negative associations. Exact ρ, p values and FDR are in Supplementary Table S2. Because SensReg is inversely associated with purity, these correlations were repeated as partial Spearman correlations adjusting for purity (n = 1,065): 12 of 22 remained significant and the five leading associations persisted with modest attenuation, whereas the regulatory-T-cell, resting CD4+ memory and M2-macrophage associations did not. (e) Representative H&E images: a SensReg-high tumor (TCGA-A1-A0SK, highly cellular, TIL-rich) and a SensReg-low tumor (TCGA-A2-A1G6, fibrotic and immune-poor); scale bars 100 μm.

**Figure 8:**
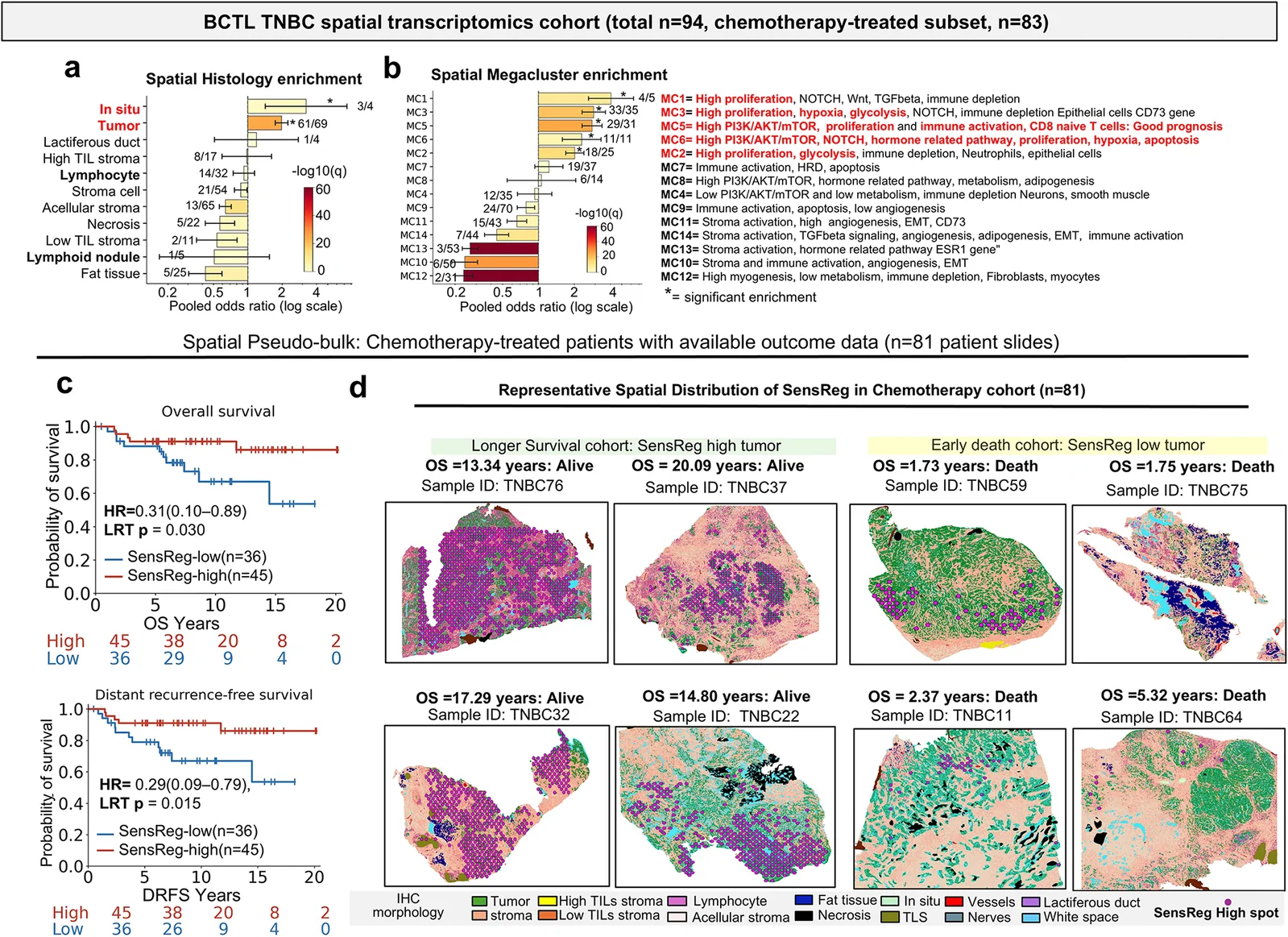
Spatial transcriptomics links SensReg-high tumor to proliferative niches, a minority of which are immune-rich, and to favorable outcome in TNBC. BCTL TNBC spatial cohort (n = 94; chemotherapy-treated subset n = 83). (a) Spatial histology enrichment across all 94 patients: odds ratio for SensReg-high versus SensReg-low spots by annotated tissue region, estimated per patient and pooled by DerSimonian–Laird random effects rather than treating nested spots as independent. Of 11 compartments, two are enriched (tumor, in situ), five depleted (fat tissue, low-TIL stroma, acellular stroma, necrosis, stroma cells) and four unchanged (lymphocyte, high-TIL stroma, lymphoid nodule, lactiferous duct). Fractions beside bars, number of patients with odds ratio >1; color, −log10 BH-q; dashed line, OR = 1. Unannotated spots and compartments present in fewer than three patients are excluded and listed in the source data. (b) Megacluster enrichment: patient-pooled odds ratio for SensReg-high spots across 14 transcriptional megaclusters (MC1–MC14; annotations at right), estimated as in (a). Five are enriched (MC1, MC2, MC3, MC5, MC6), all annotated high-proliferation; six are depleted (MC9–MC14) and three unchanged (MC4, MC7, MC8). MC1 is present in only five patients, so its leading estimate is fragile. Fractions as in (a). (c) Slide-level (spatial pseudo-bulk) Kaplan–Meier survival in chemotherapy-treated tumors with outcome data (n = 81 of 83; SensReg-low n = 36, high n = 45), for overall and distant recurrence-free survival, with profile-likelihood 95% CI and Cox likelihood-ratio p. (d) Representative longer-survival (SensReg-high) and early-death (SensReg-low) slides, colored by IHC-morphology annotation with SensReg-high spots overlaid; per-slide overall survival (years) and vital status shown.

**Figure 9:**
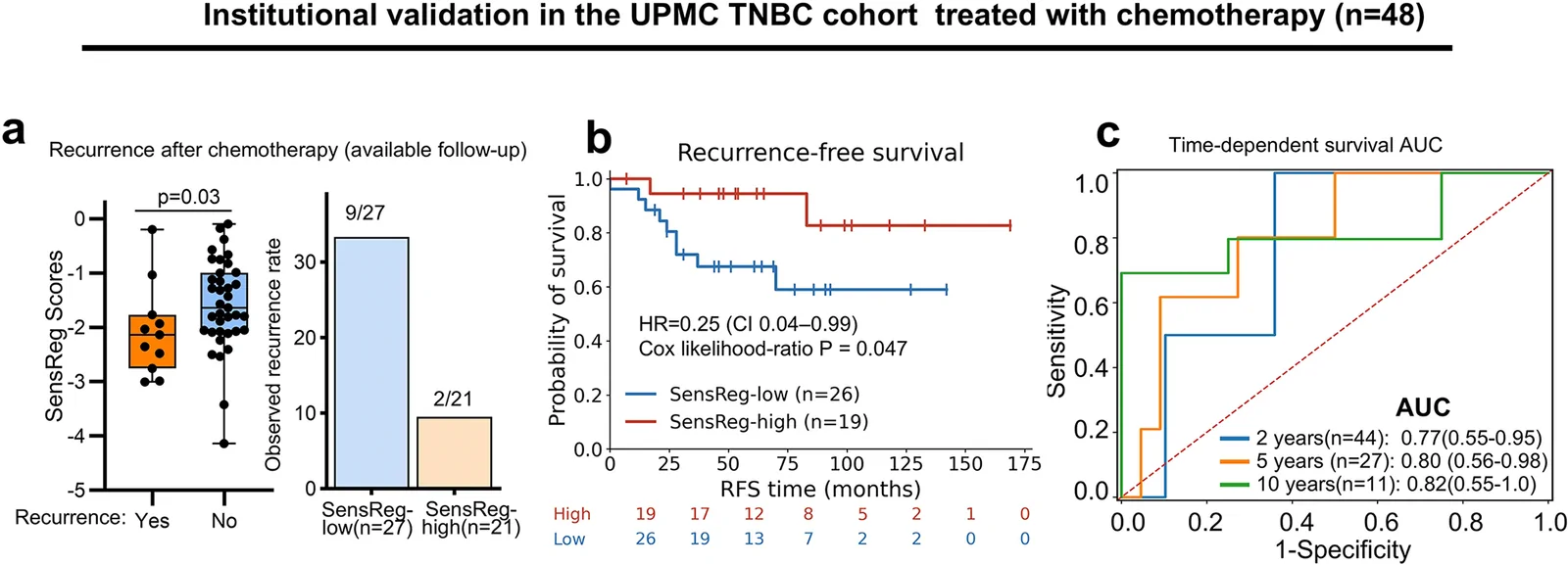
Independent institutional validation of SensReg in a published UPMC TNBC cohort. Forty-eight chemotherapy-treated patients, a subset of a previously reported series (GSE312235), were scored with the SensReg model. Median follow-up was 62 months for overall survival (range 7–169) and 65 months for recurrence-free survival (0–169). (a) Descriptive summary of observed recurrence; both sub-panels use all 48 patients and ignore differing follow-up duration. Left: recurring tumors had lower SensReg scores than non-recurring tumors during available follow-up (one-tailed Mann–Whitney U, p = 0.03; boxes median/IQR, whiskers 1.5×IQR, points individual patients). Right: observed recurrence stratified by the cohort mean SensReg score, lower in SensReg-high (2/21, 9.5%) than SensReg-low (9/27, 33%) tumors (Fisher exact one-sided p = 0.052). Because follow-up is right-censored, patients recorded as recurrence-free include those observed under 10 years; the censoring-aware comparison is panel b. (b) Recurrence-free survival by SensReg group, dichotomized at the within-cohort mean as elsewhere in this study; SensReg-high tumors had longer recurrence-free survival (HR = 0.25, 95% CI 0.04–0.99; Cox likelihood-ratio p = 0.047). (c) Time-dependent survival AUC at 2 years (0.77, 95% CI 0.55–0.95; n = 44), 5 years (0.80, 95% CI 0.56–0.98; n = 27) and 10 years (0.82, 95% CI 0.55–1.00; n = 11); intervals are wide, particularly at 10 years. For ROC calculation only, SensReg was reversed so the analysis marker increases with mortality risk; higher original scores indicate lower risk and longer survival. Diagonal, random discrimination.

## Data Availability

The UPMC TNBC expression dataset has been previously described and is available in GEO under accession GSE312235

## Code availability:

All analyses were performed using publicly available R and Python packages as detailed in Methods. Custom scripts supporting the findings of this study are available at https://github.com/wangxlab/SensReg
